# Optimal temperature for the long-term culture of adult porcine islets for xenotransplantation

**DOI:** 10.3389/fimmu.2023.1280668

**Published:** 2023-10-13

**Authors:** Naoaki Sakata, Gumpei Yoshimatsu, Ryo Kawakami, Chikao Aoyagi, Shohta Kodama

**Affiliations:** ^1^ Department of Regenerative Medicine and Transplantation, Faculty of Medicine, Fukuoka University, Fukuoka, Japan; ^2^ Center for Regenerative Medicine, Fukuoka University Hospital, Fukuoka, Japan

**Keywords:** islet transplantation, porcine, xenotransplantation, long-term culture, pancreatic stellate cell

## Abstract

Porcine islet xenotransplantation represents a promising therapy for severe diabetes mellitus. Long-term culture of porcine islets is a crucial challenge to permit the on-demand provision of islets. We aimed to identify the optimal temperature for the long-term culture of adult porcine islets for xenotransplantation. We evaluated the factors potentially influencing successful 28-day culture of islets at 24°C and 37°C, and found that culture at 37°C contributed to the stability of the morphology of the islets, the proliferation of islet cells, and the recovery of endocrine function, indicated by the expression of genes involved in pancreatic development, hormone production, and glucose-stimulated insulin secretion. These advantages may be provided by islet-derived CD146-positive stellate cells. The efficacy of xenotransplantation using islets cultured for a long time at 37°C was similar to that of overnight-cultured islets. In conclusion, 37°C might be a suitable temperature for the long-term culture of porcine islets, but further modifications will be required for successful xenotransplantation in a clinical setting.

## 1 Introduction

Pancreatic islet transplantation is a promising therapy for patients with severe diabetes mellitus (DM) and a lack of glucose control. However, this approach is limited by the size of the donor pool (1); therefore, alternative sources of islets are being evaluated, and the adult pig is considered to be an ideal donor. The adult porcine pancreas is similar to the human pancreas in size and contains a large enough number of islets to treat patients with diabetes. In addition, pigs can be readily bred to produce animals of an appropriate size and number. Porcine-specific carbohydrate antigen (2–6) and the possibility of zoonosis (7, 8) are regarded as major challenges to the use of porcine islet xenotransplantation in the clinic. However, recent progress with gene-editing technology may permit the creation of porcine-specific antigen and porcine-derived pathogen-free pigs (9, 10). Such technological progress increases the feasibility of porcine islet xenotransplantation.

For the success of adult porcine islet xenotransplantation, large numbers of high-quality islets must be obtained. However, porcine islet isolation is technically difficult, because of the vulnerability of islets, compared with islet isolation for other species (11, 12). Previous studies have shown that the expression of collagen is low in the peripheral regions of porcine islets (13), and the basement membranes of porcine islets are easily damaged during routine islet isolation (14). This fragility contributes to the difficulty of obtaining sufficient high-quality islets and culturing and maintaining them.

The establishment of a porcine islet bank, in which a large number of porcine high-quality islets can be stockpiled and accessed on demand, is a pivotal challenge in the establishment of this therapy. For this purpose, the development of a suitable method for the long-term culture of porcine islets that can maintain their viability and function is essential. Long-term culture harbors some merits in increase of the purity of islets and reduction of immunogenicity, which might contribute to the engraftment of porcine islets (15). Previous studies have attempted to characterize the effects of long-term culture on porcine islets and to determine the most appropriate conditions (16, 17). However, detailed knowledge of the effects of long-term culture on porcine islets and the mechanisms involved is still lacking. Therefore, in the present study, we aimed to characterize the effects of long-term culture on porcine islets and identify the optimal temperature for these subcellular structures, to help establish porcine islet xenotransplantation as a viable therapy.

## 2 Materials and methods

### 2.1 Study approval

The care of the animals and the experimental procedures complied with the principles of laboratory animal care (Guide for the Care and Use of Laboratory Animals, 8th edition (National Research Council, 2011)), and the experimental protocol was approved by the Animal Care and Use Committee of Fukuoka University (approval number: 2114119).

### 2.2 Study design

**Figure 1:**
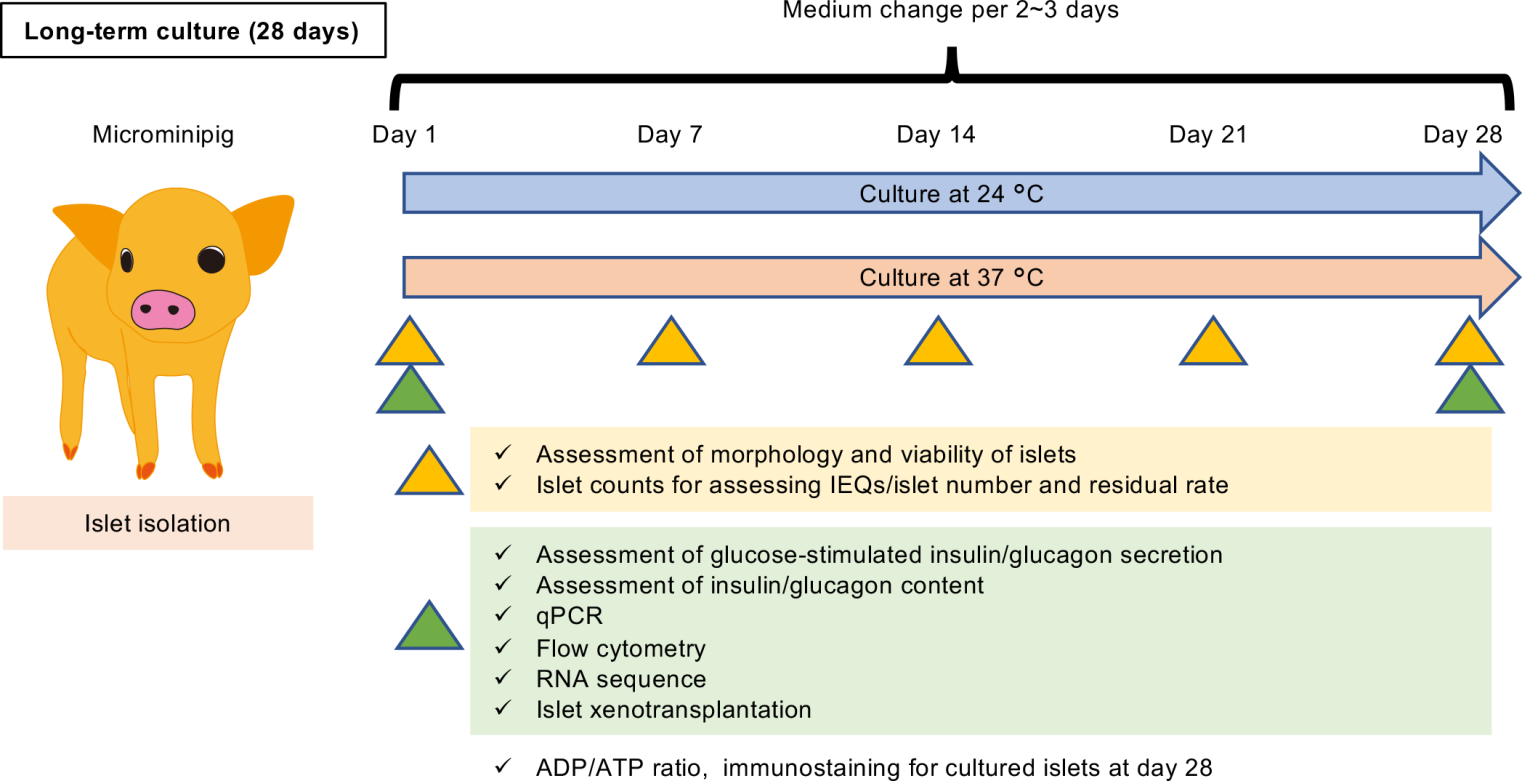
Scheme of this study design. Porcine islets isolated from each microminipig (P112, P114, P116, P117, P118, P119, P120; **Supplemental Table 1**) were cultured for 28 days by different temperature conditions, 24 or 37°C. On Day 1, 7, 14, 21, and 28, some islets were sampled and used for assessment of morphology and viability of islets. Islet counts for assessing islet equivalents (IEQs)/islet number and residual rate were also performed on the days. Sampling for assessment of glucose-stimulated insulin/glucagon secretion, assessment of insulin/glucagon content, qPCR, flow cytometry, and RNA sequence was performed on Day 1 and 28. Islet xenotransplantation using diabetic nude mice also done on the same days. Medium was changed per 2 – 3 days.

The scheme of this study design is shown in **Figure 1**. In brief, porcine islets isolated from each microminipig (P112, P114, P116, P117, P118, P119, P120; **Supplemental Table 1**) were cultured for 28 days by different temperature conditions, 24 or 37°C. On Day 1, 7, 14, 21, and 28, some islets were sampled and used for assessment of morphology and viability of islets. Islet counts for assessing islet equivalents (IEQs)/islet number and residual rate were also performed on the days. Sampling for assessment of glucose-stimulated insulin/glucagon secretion, assessment of insulin/glucagon content, qPCR, flow cytometry, and RNA sequence was performed on Day 1 and 28. Islet xenotransplantation using diabetic nude mice also done on the same days. Medium was changed per 2 – 3 days.

### 2.3 Animals

Microminipigs (https://fujimicra.co.jp/eng/product.html#whats; Fuji Micra Inc., Fujinomiya, Japan ) weighing approximately 25–30 kg were used as donor animals. The age of these pigs was 2 – 3 years at islet isolations. Male BALB/c-nu mice (https://www.clea-japan.com/products/immunodeficiency/item_a0010; CLEA Japan Inc., Tokyo, Japan) aged 8–12 weeks were the diabetic recipients. The information of microminipigs is shown in **Supplemental Table 1**. These animals were housed under specific pathogen-free conditions and had free access to food and water.

### 2.4 Procurement of pancreata

Total pancreatectomy for organ procurement was performed under general anesthesia using isoflurane (Cat#095-06573; Fujifilm Wako Pure Chemical Co., Osaka, Japan). After laparotomy, an Argyle Salem Sump tube (Covidien Japan Inc., Tokyo, Japan) was inserted into the aorta, ligated in place, and used for heparinization by the intravenous injection of heparin sodium (400 IU/kg, Cat#873334; AY Pharmaceuticals Co., Tokyo, Japan). Subsequently, the pigs were exsanguinated by incising the vena cava in the thoracic cavity, and Belzer UW^®^ Cold Storage Solution (https://amn.astellas.jp/content/dam/jp/amn/jp/ja/di/pdf/blz/Belzer_UW_Cold_Storage_Solution.pdf; Preservation Solutions, Inc. Elkhorn, WI) was infused via the tube while the abdominal organs were cooled using crushed ice. After the flushing of the circulation was completed, total pancreatomy was performed. An 18–24-gauge intravenous catheter (size according to the diameter of the pancreatic duct) was inserted into the pancreatic duct, and cold preservation solution (Cat#035-13121-2; ET-Kyoto solution; Otsuka Pharmaceutical Factory, Inc., Naruto, Japan and ulinastatin; Cat#3999405A2077; Mochida Pharmaceutical Co., Tokyo, Japan) was infused at 1 mL/g pancreas mass.

### 2.5 Porcine islet isolation and purification

A collagenase solution containing liberase MTF (0.5 g per 1 vial) and thermolysin (15 mg per 1 vial) (Cat#05339880001; Roche CustomBiotech, Penzberg, Germany) was instilled into the disinfected pancreas via the catheter placed in the pancreatic duct. The distended pancreas was cut into several pieces and then placed into a Ricordi Chamber. The digestion was started by commencing the gentle shaking of the Ricordi chamber, while warmed collagenase solution was circulated. When the digestion was stopped, the digested tissue was diluted in RPMI 1640 solution (Cat#11875085; Gibco^™^) containing 10% inactivated plasma (Fetal Bovine Serum, qualified, United States, Cat#26140079; Gibco^™^) and ulinastatin and then collected in Belzer UW^®^ Cold Storage Solution. The purification process was performed using IBM 2991 (COBE 2991; Terumo BCT, Tokyo, Japan) by centrifugation with a continuous density gradient between 1.077 g/cm^3^ and 1.100 g/cm^3^ created using Optiprep (Cat#ST-07820; Veritas Co., Tokyo, Japan). After centrifugation, the gradient density solutions containing highly-purified islets (≥ 70%) were collected. The purity was determined using the percentage of the total number of cell clusters staining positive for dithizone (Cat#D5130; Sigma-Aldrich, St. Louis, MO, USA).

### 2.6 Islet culture conditions

Islets were cultured in CMRL1066 solution (Cat# 99-603-CV; Corning, Corning, NY, USA) containing 10% fetal bovine serum (FBS), 1% antibiotics, and 200 units/L rapid insulin agent and a 5% CO_2_ atmosphere. Isolated islets were cultured at 24°C overnight and then at 24 or 37°C with 5% CO_2_ atmosphere for a further 27 days (**Figure 1A**). Information regarding the donor microminipigs and isolated islets is provided in **Supplemental Table 1**.

### 2.7 Assessment of islet number and viability

Islet number was assessed using two methods: counts and IEQs. Islet number was defined as the number of cultured islets, and IEQ was defined as the number of 150 µm-diameter islets for the normalization of islet volume (18).

To assess the viability of islets, isolated islets were stained with Hoechst^®^ 33342 (Cat#H1399; Invitrogen^™^) and propidium iodide (PI) (Cat#P1304MP; Invitrogen^™^) and the percentages of viable cells in each islet were calculated using the following formula: ([Hoechst^®^ 33342 stained cells] − [PI stained cells])/[Hoechst^®^ 33342 stained cells] × 100 (%).

Islet number, IEQ, and IEQ/islet number ratio, indicative of the mean size of the islets, and viability were measured 1, 7, 14, 21, and 28 days after islet isolation. Furthermore, morphology, assessed using islet quality score, and the residual percentage of the cells in the cultured islets were also assessed at these time points. The sums of the scores for islet shape (flat at 0 point, moderate at 1 point, oval or round at 2 point); surface (rough at 0 point, moderate at 1 point, smooth at 2 point); damage (fragmented at 0 point, moderate at 1 point, free at 2 point); the number of single cells in the culture medium (numerous at 0 point, moderate at 1 point, a few at 2 point); and the diameters of the islets (all islets <100 µm at 0 point, a few islets >200 µm at 1 point, over 10% of islets >200 µm at 2 point) were used to assess morphology (**Supplemental Table 1**). The percentage of residual islets was calculated as the percentage of cultured IEQs per IEQ 1 day following islet isolation.

### 2.8 Glucose-stimulated insulin and glucagon secretion

Glucose-stimulated insulin and glucagon secretion (GSIS and GSGS) were measured using 300 IEQs. Islets were preincubated with 3.3 mM glucose for 60 minutes, after which they were stimulated with glucose at concentrations of 3.3 mM (low glucose) or 16.5 mM (high glucose) for 60 minutes using cell culture inserts (Millicell Hanging Cell Culture Insert, PET 8 µm, 24-well; Cat#PTEP24H48; Merck Millipore, Tokyo, Japan). The porcine insulin and glucagon concentrations in the culture media were measured using an ELISA (LBIS Porcine Insulin ELISA Kit; Cat#AKRIN-013T and Glucagon ELISA Kit; Cat# 292­80001; Fujifilm Wako Shibayagi Co., Shibukawa, Japan). The absorbance at 450 nm (optical density, OD450) was determined using an iMark^™^ Microplate Absorbance Reader with Microplate Manager^®^ Software 6 (Bio-Rad, Hercules, CA, USA).

### 2.9 Measurement of insulin and glucagon concentrations

Insulin and glucagon were extracted from 300 IEQs using 1 mL RIPA buffer (Cat#16488-34; Nacalai Tesque, Kyoto, Japan) containing ×100 protease and phosphatase inhibitor cocktails (Cat#07575-51 and Cat#07574-61; Nacalai Tesque). The insulin and glucagon concentrations were measured using an LBIS Porcine Insulin ELISA Kit and a Glucagon ELISA Kit (Wako), respectively.

### 2.10 Differentiation assay for attached cells

The cells that attached during culture at 37°C were detached by incubation with TrypLE^™^ Express (Cat#12605010; Gibco^™^) for 25 minutes, collected, and seeded into wells of a 24-well plate (5×10^4^ cells/well). They were incubated in medium containing β-glycerophosphate, dexamethasone, and ascorbate for osteoblasts; or insulin, indomethacin, isobutylmethylxanthine, and dexamethasone for adipocytes (Cat#BMK-R006, Cat#BMK-R007, Cat#BMK-R008, Cat#BMK-R008; Bio future Technology, Tokyo, Japan) for over a week. The extent of differentiation into osteoblasts or adipocytes was assessed using Alizarin Red S and Oil Red O staining, respectively.

### 2.11 ELISA for transforming growth factor β1

The concentrations of TGF-β1 secreted into the medium by attached cells were measured using Human/Mouse/Rat/Porcine/Canine TGF-β1 Quantikine ELISA Kits (Cat#DB100B; R&D Systems, Inc., Minneapolis, MN, USA), in accordance with the manufacturer’s instructions.

### 2.12 ADP/ATP ratio

The ADP and ATP contents of cultured islets 28 days after isolation were measured using an ADP/ATP Ratio Assay Kit-Luminescence (Cat#346-09911; Dojindo Laboratories, Mashiki, Japan), and the ADP/ATP ratio was calculated. The absorbance at 450 nm for both ADP and ATP was measured using Spark^™^ 10M multimode microplate reader (Tecan Ltd., Männedorf, Switzerland).

### 2.13 Real-time reverse transcription polymerase chain reaction analysis

RNA was extracted from porcine islet samples using TRIzol Reagent (Cat#15596026; Invitrogen) and purified using a PureLink^®^ RNA Mini Kit (Cat#12183018A; Thermo Fisher Scientific, Waltham, MA, USA), according to the manufacturer’s instructions. The mRNA concentrations were equalized using a NanoDrop 2000 spectrophotometer (Thermo Fisher Scientific, Inc.). Reverse transcription was performed using a QuantiTect Reverse Transcription Kit (Cat#205311; Qiagen K.K., Tokyo, Japan). qRT-PCR analysis was performed using a CFX Connect Real-Time PCR Detection System (Bio-Rad Laboratories, Inc., Hercules, CA, USA) and a Thunderbird SYBR qPCR Mix (Cat#QPS-101: Toyobo Co., Ltd., Osaka, Japan). The primers used for real-time RT-PCR are shown in **Supplemental Table 2**. These were designed by Fasmac Co., Ltd. (Atsugi, Japan). Relative quantitation was performed using LightCycler Software Version 4.1 and the results were normalized to the expression of a reference gene (*Actb*). The data are presented as a fold difference, calculated using the 2^−ΔΔCt^ method.

### 2.14 RNA sequencing

The extraction, purification, and standardization of RNA extracted from cultured islets (on Days 1 and 28, after culture at 24°C or 37°C) were performed as described above. RNA sequencing libraries were prepared from 1 μg of RNA using a TruSeq Stranded mRNA LT Sample Prep Kit (Cat#20020595; Illumina), as per the manufacturer’s instructions. Cluster amplification and 151-bp paired-end sequencing were performed in accordance with the manufacturer’s protocol for NovaSeq (Illumina).

RNA sequencing was performed in Cell Innovator (Fukuoka, Japan). Read quality analysis was performed on the raw data using FastQC v0.11.7 (http://bioinformatics.babraham.ac.uk/projects/fastqc/). Quality trimming and adapter clipping were performed using Trimmomatic version 0.38 (19): trailing bases were trimmed if below the mean quality of 15, to a minimum length of 36 bases, and to remove the Illumina adapters. Trimmed reads were mapped to transcripts in the reference data for the Sus scrofa (pig) genome Sscrofa11.1 using the Bowtie2 aligner within RNA-Seq by Expectation-Maximization (RSEM) (20). The abundance of both genes and isoforms was estimated using RSEM in transcripts per million (TPM) counts.

Differentially expressed genes (DEGs) were identified using the edgeR program (21). Normalized counts per million (CPM) values, log fold-changes (logFC), and p-values were obtained from the gene-level TPM counts. The criteria for DEGs were p ≤ 0.05 and ratios ≥2 fold for upregulated genes.

### 2.15 Flow cytometry analysis

Porcine islets were dispersed to generate single islet cells using accutase (Cat#12679-54: Nacalai Tesque). These were washed in Hanks’ buffer solution containing 10% bovine serum albumin, incubated with a blocking solution, and then incubated with a primary antibody (rabbit anti-insulin (Cat#ab46716, RRID: AB_881326; 1:50; Abcam), mouse anti α-Gal Epitope (Gal alpha1–3 Gal beta1–4 GlcNAc-R) (Cat#ALX-801-090-1, RRID : AB_2111596; 1:5; Enzo Life Sciences, Inc., Lausen, Switzerland), mouse anti-pig SLA Class I (Cat#MCA2261GA, RRID : AB_324753; 1:10; Bio-Rad Laboratories, Inc.), mouse anti-pig SLA Class II DQ (Cat#MCA1335GA, RRID : AB_322326; 1:10; Bio-Rad Laboratories, Inc.), rabbit anti-CD146 antibody (Cat#ab75769, RRID : AB_2143375; 1:50; Abcam), mouse anti-smooth muscle actin (Cat#ATGA0358; 1:50; NKMAX, Seongnam, Gyeonggi, Republic of Korea), mouse anti-Ki-67 (Cat#F078801, RRID : AB_578672; 1:50; Dako (Agilent), Santa Clara, CA, USA), purified mouse IgM, κ Isotype Ctrl (Cat#401601; RRID : AB_2935847; 1:50; BioLegend, San Diego, CA, USA), purified mouse IgG1, κ Isotype Ctrl (Cat#401402, RRID : AB_2801451; 1:5; BioLegend), or purified rabbit polyclonal Isotype Ctrl (Cat#910801, RRID : AB_2722735; 1:43; BioLegend)). Donkey anti-mouse IgG A647 (Cat#ab150107, RRID : AB_2890037; 1:1,000; Abcam) and donkey anti-rabbit IgG A488 (Cat#ab98488, RRID : AB_10676096; 1:1,000; Abcam) secondary antibodies were used. Fixation/Permeabilization Solution Kit (Cat#554714; BD, Franklin Lakes, NJ, USA) was used for intracellular flow cytometry. Flow cytometry was performed using a BD Accuri^™^ C6 Plus flow cytometer (BD).

### 2.16 Induction of diabetes in recipient mice

Diabetes was induced in recipient mice by the intravenous injection of streptozotocin (220 mg/kg body mass; Cat#S0130; Sigma-Aldrich). Mice with blood glucose concentrations exceeding 400 mg/dL were used as diabetic recipients.

### 2.17 Islet transplantation

Recipient mice were anesthetized using isoflurane, then a dorsal incision was made through the muscle and peritoneum and the left kidney was mobilized outside the abdomen. The renal capsule was peeled off from the parenchyma to prepare the renal subcapsular space for the transplantation of islets. Overnight or 28-day-cultured porcine islets were placed into the space using Gastight Syringes 1002 RN (Hamilton Company Inc., Reno, NV, USA) and Intramedic polyethylene tubing 0.58 mm (Cat#BD427410; Becton Dickinson, Franklin Lakes, NJ, USA). After transplantation, the kidney was replaced into the abdomen and the incision was sutured.

### 2.18 Assessment of the function of transplanted islets

The function of the transplanted islets was assessed by monitoring the blood glucose and plasma porcine C-peptide concentrations. Normoglycemia was defined as a blood glucose concentration of <200 mg/dL. The plasma porcine C-peptide concentrations were measured using a Porcine C-peptide ELISA (Cat#10-1256-01; Mercodia, Winston Salem, NC, USA).

### 2.19 Histological assessment

The left kidneys of the recipient mice were dissected following euthanasia and the transplanted islets were evaluated. Cultured islets were embedded in agarose gel for the evaluation of any changes that had occurred during long-term culture. Three-micrometer-thick sections were either stained with hematoxylin and eosin (HE) or subjected to immunohistochemistry (for insulin to identify islets, for von Willebrand factor (vWF) to identify vessels, for porcine C-peptide to identify porcine islets, for mouse C-peptide to identify mouse islets, for Ki67 to evaluate cellular proliferation, for collagen I or fibronectin to identify ECM in the cultured islets, for integrin β1 or E-cadherin (adhesion factors), for CD146 or α smooth muscle actin (SMA) to identify PSCs, for PDX-1 (a marker of β cells or pancreatic progenitors), or α-Gal (a carbohydrate antigen)). The primary antibodies used were guinea pig anti-insulin (Cat#A056401-2, RRID : AB_2617169; 1:100; Agilent, Dako, Tokyo, Japan), rabbit anti-insulin (Cat#ab181547, RRID : AB_2716761; 1:1,000; Abcam, Cambridge, UK), sheep anti-glucagon (Cat#ab36232, RRID : AB_732575; 1:100; Abcam), rabbit anti-somatostatin (Cat#ab103790, RRID : AB_10711731; 1:500; Abcam), mouse anti-pig C-peptide (Cat#MAA447Po21; 1:200; Cloud-Clone Corp. MAA447Po21, Katy, TX, USA), mouse anti-mouse C-peptide (Cat#NBP1-05433, RRID : AB_1556271; 1:500; Novus Biologicals NBP1-05433, Centennial, CO, USA), rabbit anti-vWF antibody (Cat#ab179451, RRID : AB_2890242; 1:100; Abcam), rabbit anti-Ki67 antibody (Cat#ab66155, RRID : AB_1140752; 1:200; Abcam), rabbit anti-collagen I antibody (Cat#ab138492, RRID : AB_2861258; 1:500; Abcam), rabbit anti-fibronectin (Cat#ab2413, RRID : AB_2262874; 1:100; Abcam), rabbit anti-integrin β1 (Cat#ab179471, RRID : AB_2773020; 1:1,000; Abcam), rabbit anti-E cadherin (Cat#ab40772, RRID : AB_731493; 1:500; Abcam), rabbit anti-CD146 (1:200; Abcam), rabbit anti-αSMA (Cat#ab15734, RRID : AB_443242; 1:200; Abcam), mouse anti-PDX1 (Cat#sc-390792, RRID : AB_2938928; 1:100; SantaCruz), and mouse anti-α-Gal (1:5; Enzo Life Sciences, Farmingdale, NY, USA). After incubation with a primary antibody, donkey anti-mouse IgG (H+L) Alexa488 (Cat#715-547-003, RRID : AB_2340851; 1:100; Jackson ImmunoResearch Laboratories, Inc., West Grove, PA, USA), Alexa 488-conjugated donkey anti-guinea pig (Cat#715-547-003, RRID : AB_2340472; 1:100; Jackson ImmunoResearch Laboratories, Inc.), Alexa Fluor^®^ 488 AffiniPure goat anti-rat IgG (H+L) (Cat#112-545-003, RRID : AB_2338351; 1:100; Jackson ImmunoResearch Laboratories, Inc.), Alexa Fluor^®^ 647 AffiniPure goat anti-rabbit IgG (H+L) (Cat#111-605-144, RRID : AB_2338078; 1:100; Jackson ImmunoResearch Laboratories, Inc.), Cy3-conjugated goat anti-rabbit (Cat#111-165-144, AB_2338006; 1:100; Jackson ImmunoResearch Laboratories, Inc.), Alexa 488 anti-goat, Alexa 647 anti-goat, Alexa 647 anti-mouse, and Cy3 anti-goat were used as secondary antibodies. Nuclear staining was performed using 4′,6-diamidino-2-phenylindole (DAPI: Cat#340-07971; Dojindo). Histological images were obtained using a BZ-X700 microscope (Keyence, Itasca, IL, USA) and immunostaining was quantified using ImageJ^®^ software (https://imagej.nih.gov/ij/index.html; National Institutes of Health, Bethesda, MD, USA).

### 2.20 TUNEL assay

TdT-mediated dUTP nick end labeling (TUNEL) assay was performed to long-term cultured islets using TACS2 TdT *in situ* Apoptosis Detection Kit-Fluorescein (Cat# 4812-30-K; R&D Systems), to detect apoptotic islet cells. Double staining for insulin, glucagon or somatostatin was done to the same specimen for detecting apoptotic cells in β, α, δ cells, respectively.

### 2.21 Statistical analysis

The blood glucose and plasma C-peptide concentrations and the changes in blood glucose concentration during glucose tolerance testing were compared using two-way repeated measures analysis of variance, followed by Dunnett’s test, as appropriate. Data are presented as the mean ± standard error of the mean. *p* < 0.05 was used to define statistical significance. All tests were two-sided. Statistical analyses were conducted using JMP^®^12.0.0 (SAS Institute Inc., Cary, NC, USA).

## 3 Results

### 3.1 Culture at 37°C stabilizes the morphology and promotes the cellular proliferation of long-term cultured porcine islets

**Figure 2:**
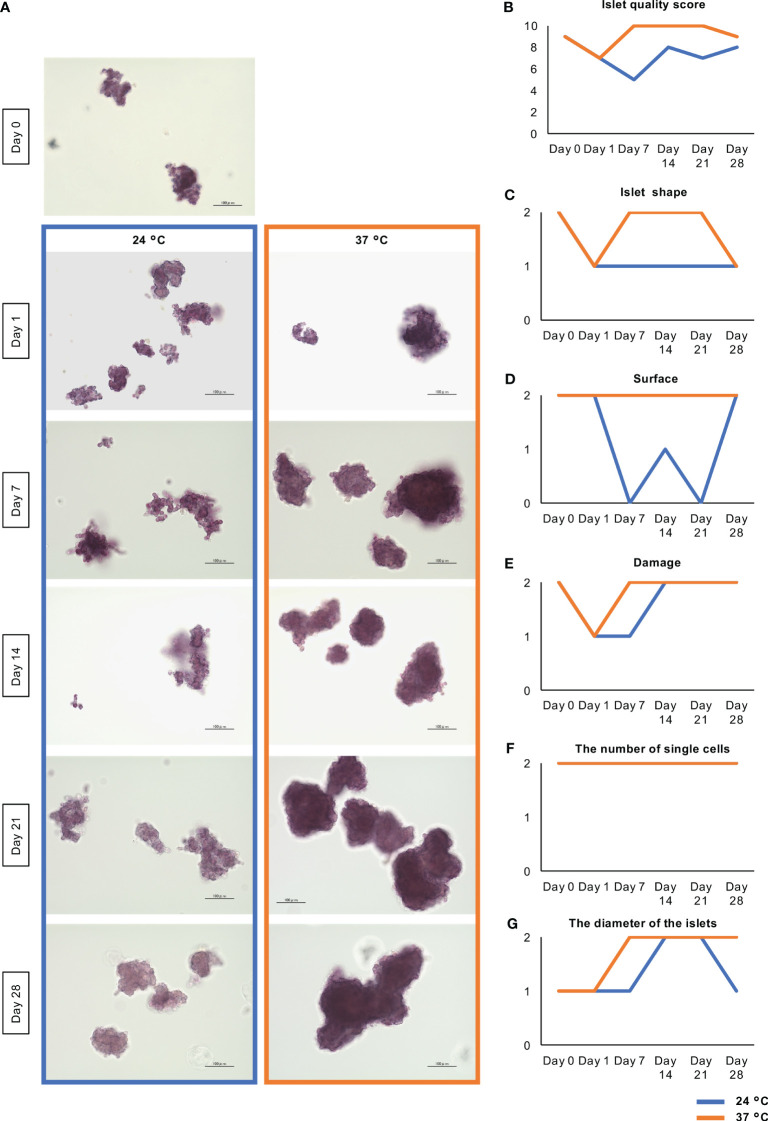
Morphological change of long-term cultured porcine islets. **(A)** Dithizone-stained islets isolated from one pig cultured at 24°C (blue) and 37°C (orange) on Days 0, 1, 7, 14, 21, and 28. Most of the islets cultured at 37°C became solid with a smooth surface throughout the observation span. On the other hand, most islets cultured at 24°C had a rough and frayed surface. Scale bar: 100 µm. **(B)** Change of the islet quality score. This score is composed of he sums of the scores for islet shape (flat at 0 point, moderate at 1 point, oval or round at 2 point); surface (rough at 0 point, moderate at 1 point, smooth at 2 point); damage (fragmented at 0 point, moderate at 1 point, free at 2 point); the number of single cells (numerous at 0 point, moderate at 1 point, a few at 2 point); and the diameters of the islets (all islets <100 µm at 0 point, a few islets >200 µm at 1 point, over 10% of islets >200 µm at 2 point). **(C-G)**. Change of the parameters for calculating islet quality scores including islet shape **(C)**, surface **(D)**, damage **(E)**, the number of single cells **(F)**, and the diameter of the islets **(G)**.

First, we aimed to characterize long-term (28-day) cultured islets. **Figure 2** shows the morphological changes of the islets during the long-term culture. On Day 0 (i.e. at preculture), the porcine islets in this assay accompanied with round shape (islet shape: 2 point) and smooth surface (surface: 2 point). There were no damaged islets (damage: 2 point) with few dispersed single cells (the number of single cells: 2 point). On the other hand, a few over 200 µm-sized islets were seen (the diameters of the islets: 1 point). The islet quality score of these islets was 9 points (**Figures 2A, B**). On Day 1, the islet quality score was declined to 7 points because the shape of islets became flat and fragmented islets were moderately shown (**Figures 2A, B, C, E**). After that, the surface of the cultured islets became smooth over time, especially those cultured at 37°C. Most of the islets cultured at 37°C became solid, with a smooth surface, between days 7 and 14 (**Figure 2A**). The islet quality score was 10 points during the span (**Figure 2B**). On the other hand, most islets cultured at 24°C had a rough and frayed surface at these time points (**Figure 2A**). The islet quality score was lower comparing with islets cultured at 37°C during the observation span (**Figure 2B**). Final islet quality score on Day 28 was 9 points at 37°C and 8 point at 24°C. Score in islet shape, surface, damage and the diameter of the islets was exceeded in 37°C, while there were no change in the number of single cells (**Figures 2C–G**).

**Figure 3:**
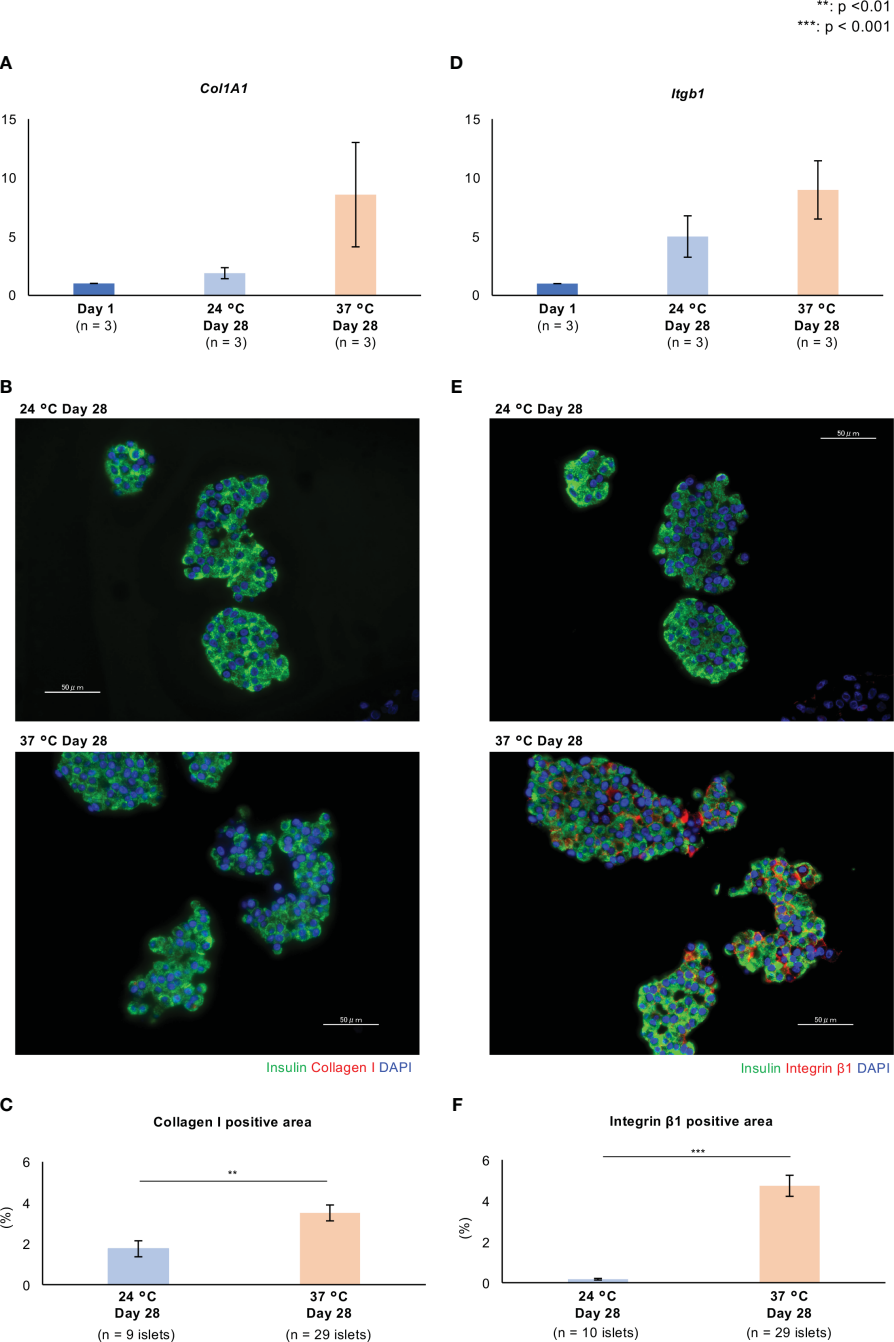
Expression of extracellular matrix proteins and adhesion factors in long-term cultured porcine islets. **(A, D)**. Expression of *Col1a1* **(A)** and *Itgb1* **(D)** in cultured porcine islets. *Col1a1* and *Itgb1* encode collagen I and integrin β1, respectively. The ratios of the expression between Day 1 and other culture conditions (24°C Day 28 and 37°C Day 28), quantified using the 2^−ΔΔCt^ method. n = 3 islet isolations. **(B, E)**. Histology of isolated islets which were cultured at 24°C (upper) and 37°C (lower). Sections are immunostained with anti-insulin (green), anti-collagen I red in **(B)**, and anti-integrin β1 red in **(E)** antibodies. **(C, F)**. Collagen I **(C)** and integrin β1 **(F)**-positive areas per islet area. DAPI (blue) counterstaining for nuclei was used. ** *p* < 0.01, *** *p* < 0.001. Scale bar: 50 µm.

In this morphological assessment, aggregation of the islets was noticeable after 21 days of culture at 37°C (**Figure 2A**). We considered that these morphological changes might be the result of higher expression of extracellular matrix (ECM) proteins and adhesion factors that strengthen cell-to-cell junctions. As representative ECM proteins, the expression of collagen I and fibronectin in long-cultured islets was evaluated by qPCR and immunofluorescence staining. The expressions of *Col1A1*, which encodes collagen I, tended to be high in long-term cultured islets, and especially in those cultured at 37°C, while there were no significant differences among the three culture conditions (**Figure 3A**; **Supplemental Figure 1A**). Collagen I was mainly expressed on the surfaces of the endocrine cells, but the level of expression was very weak (**Figure 3B**; **Supplemental Figure 1B**). The collagen I-positive area of the long-term cultured islets was significantly larger in those cultured at 37°C than in those cultured at 24°C (*p* = 0.005; **Figure 3C**). In contrast, the expression of *Fn1*, which encodes fibronectin, was significantly lower, as was the area of the islets that was immunopositive for fibronectin (**Supplemental Figures 2A–C**).

Integrin β1 and E-cadherin are the major adhesion factors that contribute to cell-to-cell junctions. The former is a receptor for various ECMs, including collagen and fibronectin, and forms focal adhesions, multi-protein complexes that mediate contact between cells and the ECM. Integrin β1 is expressed on the surfaces of endocrine cells in mouse islets (22), and E-cadherin is expressed on cell membranes, where it interacts with similar molecules on other cells, to form cell-cell adherens junctions (23). In the present study, we found that the expression of *Intgb1*, which encodes integrin β1, in islets cultured at 37°C for 28 days was significantly higher than in islets cultured overnight and at 24°C for 28 days, in each isolation (#1 - #3 islet isolation) (37°C Day 28 vs. Day 1: *p* = 0.013, *p* = 0.001, *p* = 0.049; 37°C Day 28 vs. 24°C Day 28: *p* = 0.016, *p* = 0.040, *p* > 0.05; **Supplemental Figure 1C**; **Figure 3D**). Furthermore, the expression of integrin β1 was high in cell-to-cell junctions after culture at 37°C, and lower after culture at 24°C (*p* < 0.001; **Figures 3E, F**). The expression of *Cdh1*, which encodes E-cadherin, was higher after long-term culture, especially at 24°C (**Supplemental Figure 2D**), but the expression of E-cadherin protein was relatively low at both 24°C and 37°C (**Supplemental Figures 2E, F**). Thus, the expression of collagen I and integrin β1 following long-term culture at 37°C is consistent with an enhancement of cell-ECM junctions, which might contribute to the stability of the islets.

**Figure 4:**
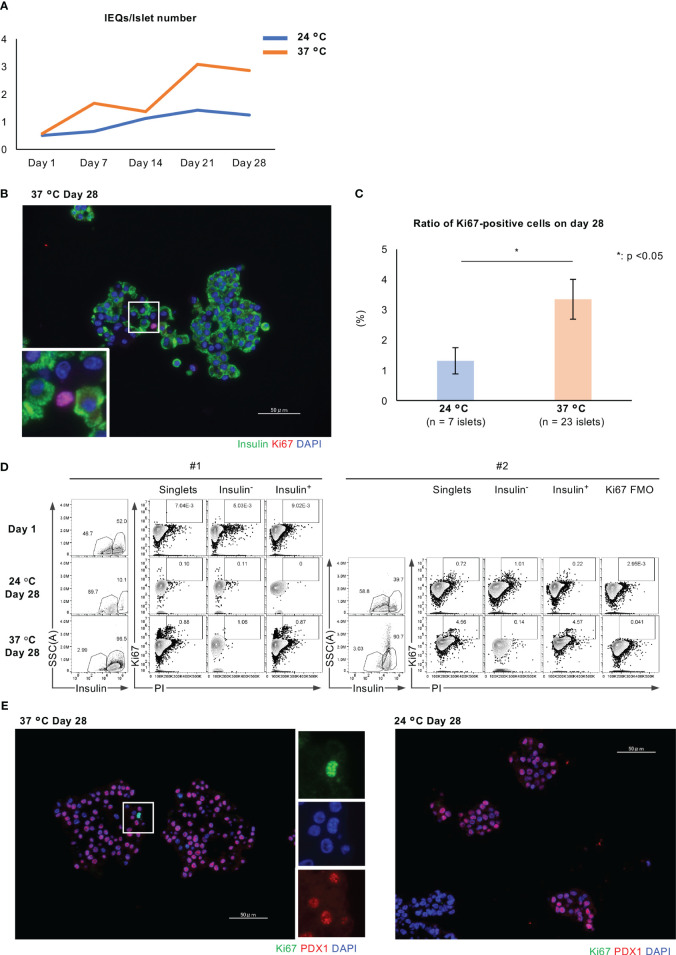
Cellular proliferation of long-term cultured porcine islets. **(A)** Change in the IEQ/islet number of long-term cultured porcine islets isolated from a pig at 24°C (blue) and 37°C (orange). IEQ/islet number indicates the average size of islets. **(B)** Histology of isolated islets which were cultured at 37°C, immunostained for insulin (green) and Ki67 (red). **(C)** Ratio of Ki67-positive islet cells per total islet cells in the islets cultured at 24°C (pale blue) or 37°C (pale orange) for 28 days. **(D)** Results of the flow cytometry analysis of Ki67-positive cells, Ki67-positive and insulin-negative cells, and insulin/Ki67 double-positive cells in islets on Day 1, after culture at 24°C on Day 28, and after culture at 37°C on Day 28. #1 and #2 means the number of islet isolations, respectively (i.e. islet isolation number 1 and 2). We had two islet isolations for these flow cytometry analyses. **(E)** Histology of long-term cultured islets at 37°C (left) and 24°C (right) on Day 28. They were immunostained for Ki67 (green) and PDX1 (red), and counterstained using DAPI (blue). * *p* < 0.05. Scale bar: 50 µm.

We also found that long-term culture was associated with an increase in islet size. The IEQ/islet number ratio, an index of the mean size of an islet, increased during long-term culture, and was higher after culture at 37°C than at 24°C (**Figures 2A**, **4A**). We considered that the larger islet size after culture at 37°C might reflect greater cellular proliferation. To elucidate the mechanism underlying this difference, we assessed the expression of Ki67, a marker of cellular proliferation, in the islets, and found few Ki67-positive cells, especially at 37°C (ratio of Ki67-positive cells: *p* = 0.02; **Figures 4B, C**). We next quantified the population of insulin/Ki67 double-positive cells in the long-term cultured islets using flow cytometry, and found that long-term culture increased the numbers of Ki67-positive cells in islets cultured at 24°C, and especially at 37°C, indicating a promotion of cellular proliferation (Insulin^−^/Ki67^+^: 0.00503% on Day 1, 0.11% at 24°C on Day 28, 1.06% at 37°C on Day 28; Insulin^+^/Ki67^+^: 0.00902% on Day 1, 0% at 24°C on Day 28, 0.87% at 37°C on Day 28 for #1 islet isolation; Insulin^+^/Ki67^+^: 0.22% at 24°C on Day 28, 4.57% at 37°C on Day 28 for #2 islet isolation; **Figure 4D**). These data indicate that the proliferation of islet cells, and especially β cells, is more highly upregulated after long-term culture at 37°C than at 24°C.

Immunofluorescence staining for Ki67 and PDX1, which is a marker of endocrine differentiation that is expressed by pancreatic progenitors and adult β cells, showed Ki67-positive and PDX-negative cells in long-term cultured islets, especially islets at 37°C (**Figure 4E**). We speculate that these cells might be not only non-β cells, but also extra-endocrine cellular components that contribute to the increase in islet size.

### 3.2 Long-term culture does not reduce the viability of porcine islets

**Figure 5:**
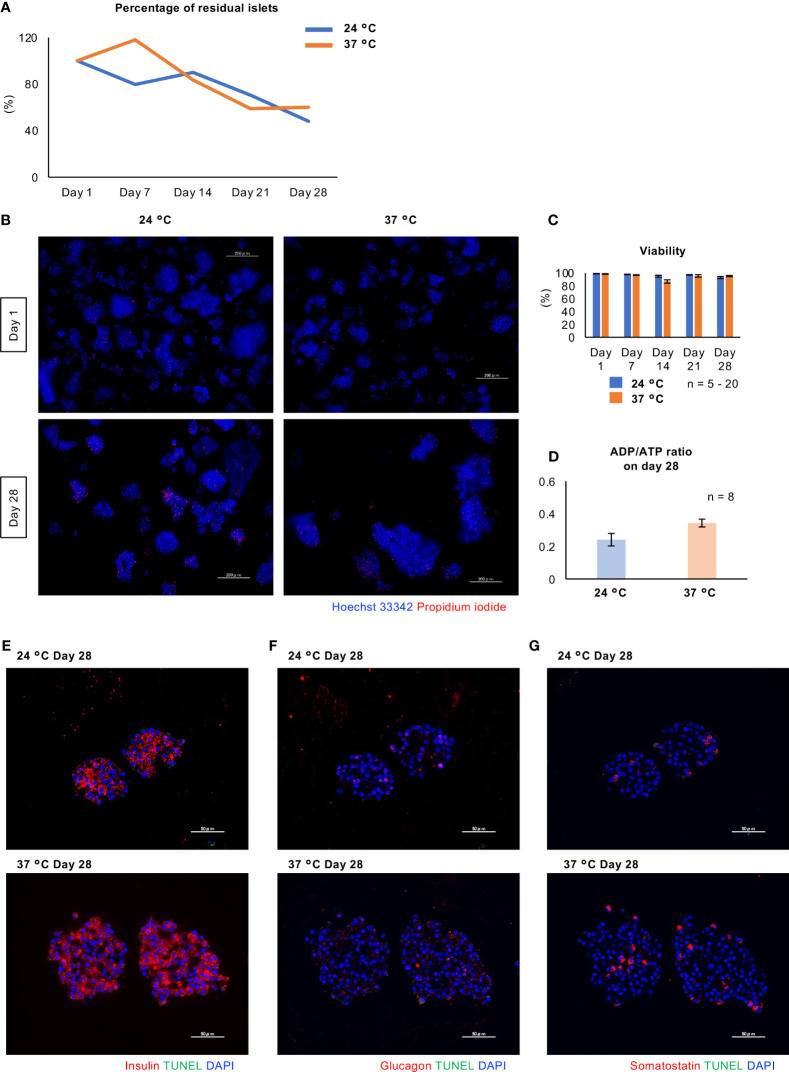
Viability of long-term cultured porcine islets. **(A)** Change of percentage of residual islets isolated from a pig during long-term culture (24°C: blue, 37°C: orange). **(B)** Cultured islets at 24°C (left) and 37°C (right), stained with Hoechst 33342 (blue) and propidium iodide (red) on Days 1 and 28. **(C)** Viability of the porcine islets during the culture. Viability was assessed by the formula ([Hoechst^®^ 33342 stained cells] − [PI stained cells])/[Hoechst^®^ 33342 stained cells] × 100 (%). **(D)** ADP/ATP concentration ratio, indicating mitochondrial damage, for the cultured islets on Day 28.(Prior to **(D)**) Scale bar: 100 µm. **(E - G) **TUNEL staining (green) for long-term cultured islets. Double staining with insulin (**E**; red), glucagon (**F**; red) and somatostatin (**G**; red). DAPI (blue) was used for counter staining. Scale bar: 50 µm.

**Figure 5** shows the viability of long-term cultured porcine islets. Regarding the percentage of residual islets (residual islet equivalents, compared to Day 1), the percentage of residual islets similarly decreased after culture at either 24°C or 37°C (**Figure 5A**). On the other hand, over 95% of the viability of the islet cells was retained during long-term culture at either temperature, with no significant difference between the two (**Figures 5B, C**). Furthermore, there was no significant difference in the ADP/ATP ratio, indicative of damage to mitochondria, between the two conditions after 28 days of culture (**Figure 5D**). We also assessed the apoptosis of long-term cultured islets, and could not detect any apoptotic cells in β, α and δ cells of the islets at both 24°C and 37°C (**Figures 5E-G**).

### 3.3 Long-term culture of porcine islets at 37°C is associated with partial recovery of endocrine function, associated with increases in the expression of genes involved in pancreatic differentiation

**Figure 6:**
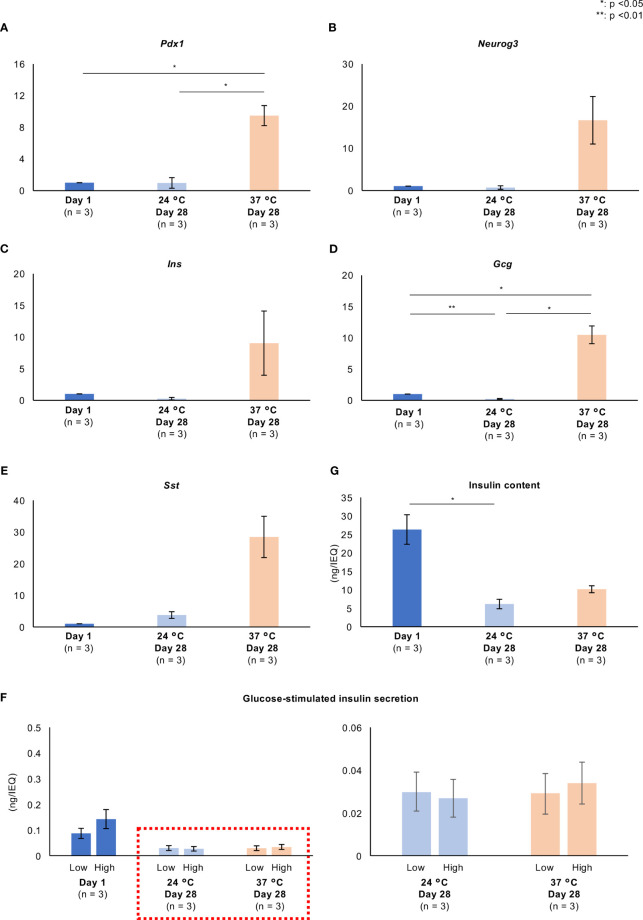
Pancreatic differentiation and endocrine function of long-term cultured porcine islets. **(A–E)**. Expression of genes involved in pancreatic differentiation **(A)**: *Pdx1*, **(B)**: *Neurog3*) and encoding hormones **(C)**: *Ins*, D: *Gcg*, E: *Sst*) in cultured porcine islets. Day 1: blue, 24°C Day 28: pale blue, and 37°C Day 28: pale orange. The ratios of the expression after 1 day and 28 days (both temperatures) are shown as 2^−ΔΔCt^ values. **(F)** Glucose-stimulated insulin secretion by cultured islets in response to low and high glucose stimulations. **(G)** Insulin content per islet. n = 3 islet isolations. * *p* < 0.05, ** *p* < 0.01.

**Figures 6A–E** and **Supplemental Figures 3A-E** show the expression of genes involved in pancreatic differentiation (*Pdx1* and *Neurog3*) and encoding hormones (*Ins*, *Gcg*, *Sst*). Long-term culture at 37°C increased the expression of all of these genes. GSIS and the insulin content of the islets were markedly reduced by 28 days of culture at either 24°C or 37°C (**Figure 6F**); however, the secretion of insulin in response to a high glucose concentration and the insulin content tended to be higher in islets cultured at 37°C than in those cultured at 24°C (**Figures 6F, G**; **Supplemental Figures 3F, G**). These data imply that long-term culture at 37°C is associated with the partial recovery of the endocrine function of porcine islets. The GSGS and glucagon content of the islets was attenuated by long-term culture (**Supplemental Figures 4A, B**).

### 3.4 Porcine islets contain multipotent stem cells that might mediate cellular proliferation and the recovery of endocrine function when cultured long-term at 37°C

**Figure 7:**
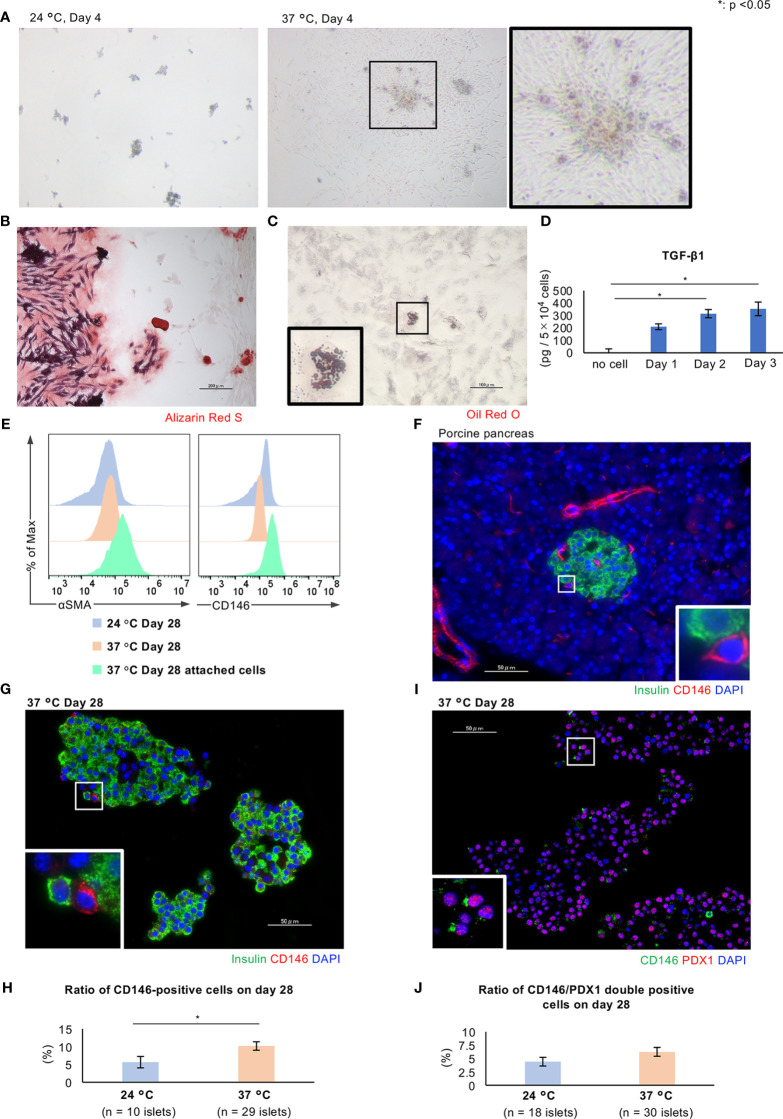
Characteristics of CD146-positive cells in long-term cultured porcine islets. **(A)** Porcine islets cultured at 24°C (left) or 37°C (center) for 4 days. Spindle cells derived from the islets attached to the bottom of the culture flask and proliferated (center and right). **(B)** Differentiation of the attached cells into osteoblasts. Alizarin Red S staining to demonstrate differentiated osteoblasts. **(C)** Differentiation of the attached cells into adipocytes. Oil red O staining to demonstrate differentiated adipocytes. **(D)** Concentration of TGF-β1 secreted by attached cells. **(E)** Results of flow cytometry analysis of the attached cells (pale green) and islets cultured at 24°C (pale blue) or 37°C (pale orange) for the detection of CD146 (right) or αSMA (left)-positive cells. **(F)** CD146-positive cells in the porcine pancreas immunostained for insulin (green) and CD146 (red). **(G)** Histology of islets cultured at 37°C, immunostained for insulin (green) and CD146 (red). **(H)** Ratio of CD146-positive cells in islets cultured at 24°C (pale blue) or 37°C (pale orange) for 28 days. **(I)** Histology of islets cultured at 37°C, immunostained for CD146 (green) and PDX1 (red). DAPI (blue) was used for counterstaining. **(J)** Ratio of CD146/PDX1-positive cells in islets cultured at 24°C (pale blue) or 37°C (pale orange) for 28 days. * *p* < 0.05. Scale bar: 50 µm.

As shown in **Figure 5A**, the percentage of residual islets similarly decreased after culture at either 24°C or 37°C. Cells derived from islets attached to and proliferated on the bottom of culture flasks at 37°C, but not at 24°C. This phenomenon trapped many islets and contributed to the reduction in percentage of residual islets (**Figure 7A**).

Although the attachment of cells caused a loss of long-term cultured islets, we hypothesized that this might provide some benefits to the islets with respect to cellular proliferation and endocrine function. Therefore, the characteristics of the islet cells that attached to the culture vessel during long-term culture at 37°C were assessed. We first evaluated the multipotency of these attached cells using a Mesenchymal Stem Cell (MSC) Identification Kit. Some of the cells were capable of differentiating into osteoblasts and inducing calcification (**Figure 7B**), or into adipocytes containing lipid droplets (**Figure 7C**). Furthermore, the attached cells produced transforming growth factor beta 1 (TGF-β1) (**Figure 7D**). Thus, the attached cells include MSCs that are multipotent and secrete substances with paracrine effects. We speculate that these cells might be pancreatic stellate cells (PSCs), which are pancreas-resident stem cells.

PSCs play a critical role in pancreatic fibrosis in chronic pancreatitis and pancreatic cancer (24) and act as multipotent stem cells, generating pancreatic β cells (25). There are several specific markers of PSCs, and we used two in the present study: CD146 and α-smooth muscle actin (SMA). CD146 is also known as melanoma cell adhesion molecule (26) and is a marker of early MSCs (27–29). CD146-positive MSCs have a high level of multipotency, and can give rise to endothelial cells, osteoblasts, chondrocytes, and adipocytes (30, 31). αSMA is also a marker of activated PSCs (32). Flow cytometry analysis of the attached cells derived from porcine islets revealed that they included CD146-positive and αSMA-positive cells, and the numbers of each were higher comparing with those in islets cultured at either 24°C or 37°C (**Figure 7E**). The immunofluorescence staining of sections of porcine pancreas revealed a few CD146-positive cells in both intra- and extra-islet tissues (**Figure 7F**), and also in long-term cultured islets, especially if they were cultured at 37°C (*p* = 0.04; **Figure 7G, H**). Some of the CD146-positive cells were also immunopositive for αSMA (data not shown). We also assessed the characteristics of the CD146-positive cells in the islets as the potential progenitors of pancreatic cells. Immunofluorescence staining of long-term cultured islets for CD146/PDX1 revealed the presence of double-positive cells, which might represent pancreatic progenitors that can differentiate into endocrine cells (**Figures 7I, J**).

### 3.5 The expression of α-Gal is not high in porcine islets

We next aimed to determine whether long-term culture reduces the immunogenicity of cultured islets because this would increase the chances of successful xenotransplantation (33). **Supplemental Figure 5A**, B shows the expression of *Ggta1p* and *Cmah*. Long-term culture reduced the expression of *Cmah* in islets cultured at 24°C (*p* = 0.0008; **Supplemental Figure 5B**). The expression of *Ggta1p* was reduced by long-term culture at 37°C (*p* = 0.0016), but the expression level was significantly higher after culture at 24°C than after culture at 37°C (*p* = 0.0028; **Supplemental Figure 5A**).

Next, we performed a flow cytometry analysis of long-term cultured porcine islets isolated from three pigs (#1 - #3 islet isolation) to further characterize the expression of α-Gal. Interestingly, no α-Gal-positive islet cells were obtained from #1 or #3 islet isolation (**Supplemental Figure 5C**), but they were identified in the islets obtained from #2 islet isolation, and there were more in the islets cultured at 37°C than at 24°C (**Supplemental Figure 5C**). Immunofluorescence staining for α-Gal did not show any α-Gal positive cells in islets cultured at either 24 or 37°C (**Supplemental Figure 5D**), but the endothelium of the abdominal artery showed strong immunostaining (**Supplemental Figure 5E**). Thus, we could not find evidence of α-Gal expression in porcine islets. The α-Gal-positivity obtained using flow cytometry might have been a pseudo-positive finding because of the high level of background staining (**Supplemental Figure 5C**).

As a further assessment of immunogenicity, we performed flow cytometry to identify cultured islet cells that expressed SLA class I or SLA class II DQ. Swine leukocyte antigens (SLAs) are porcine major histocompatibility (MHC) antigens, which are mediators of humoral rejection following transplantation (34). Both SLA classes I and II can cause the rejection of xenotransplants because they can be targeted by human leukocyte antigen-specific antibodies (35). We found that SLA class I was expressed by overnight-cultured porcine islets (Day 1) (**Supplemental Figure 5F**), and SLA class II DQ was expressed in islets from #1 islet isolation, but not in those from #3 islet isolation (**Supplemental Figure 5F**). Although the number of SLA class I-positive cells did not change, the number of SLA class II DQ-positive cells increased during long-term culture, contrary to our expectation that the expression would decrease.

### 3.6 RNA sequencing revealed that islets cultured long-term at 37°C demonstrate higher levels of insulin secretion, focal adhesion, and cellular proliferation

Sequencing of RNA extracted from porcine islets and cultured overnight, or at 24°C or 37°C for 28 days, followed by data analysis using the Database for Annotation, Visualization and Integrated Discovery (DAVID: https://david.ncifcrf.gov) was next performed to further characterize the effects of long-term culture. We found that the expression of genes involved in ion transport (*Kcnip1*, *Calb1*), cell differentiation (*Ush2a*, *Th*, *Fev*), and the downregulation of apoptosis (*Scg2*) was higher in islets cultured for 28 days at 37°C (**Table 1**) than in those cultured at 24°C. We then used the Kyoto Encyclopedia of Genes and Genomes (KEGG) pathway classification to interrogate the functions of the upregulated genes. A comparison with 24°C-cultured cells on Day 28 showed that “Metabolic pathway”, “Protein processing in endoplasmic reticulum”, and “Insulin secretion” were upregulated in the 37°C-cultured cells (**Table 2**). Specifically, genes involved in insulin secretion, ion transport, including the calcium signaling pathway, and the cAMP signaling pathway were upregulated at 37°C. We hypothesized that these differences might have been induced by a thermosensitive system that included transient receptor potential (TRP) channels, and therefore measured the expression of genes encoding these channels. Of these, *Trpm5* (log-fold difference 5.09, *p* = 1.45E−44) was found to be upregulated in cells cultured for 28 days at 37°C.

**Table 1:** The 20 most upregulated genes in islets cultured for a long period at 37°C, compared with 24°C.

log fold-changes, p-value	Gene symbol and name	Accession number
7.16 4.76E-30	SLIT1 (Slit guidance ligand 1)	XM_021072814
7.12 1.92E-29	CAPS (Calcyphosine)	NM_001244975
6.89 5.46E-144	SCG2 (Secretogranin II)	NM_001012299
6.76 1.98E-24	ST8SIA2 (ST8 alpha-N-acetyl-neuraminide alpha-2,8-sialyltransferase 2)	NM_001315676
6.44 1.14E-20	USH2A (Usherin)	XM_021064292
6.33 2.10E-73	MOXD1 (Monooxygenase DBH like 1)	XM_001926931
6.29 9.83E-121	HEPACAM2 (HEPACAM family member 2)	XM_003357438
6.05 1.40E-71	KCNIP1 (Potassium voltage-gated channel interacting protein 1)	NM_001031777
5.86 4.81E-23	PRODH2 (Proline dehydrogenase 2)	XM_021097109
5.84 2.32E-114	TH (Tyrosine hydroxylase)	XM_021085452
5.84 2.77E-48	DPEP2 (Dipeptidase 2)	XM_003355779
5.59 3.69E-60	GRIK1 (Glutamate ionotropic receptor kainate type subunit 1)	XM_003358905
5.59 1.17E-12	FEV (FEV transcription factor, ETS family member)	XM_021075453
5.55 2.33E-72	DGKB (Diacylglycerol kinase beta)	XM_021102437
5.54 3.66E-82	CALB1 (Calbindin 1)	NM_001130226
5.45 1.92E-36	GALNTL6 (Polypeptide N-acetylgalactosaminyltransferase like 6)	XM_021072439
5.37 5.91E-44	LDHD (Lactate dehydrogenase D)	XM_021093784
5.20 1.96E-20	CLVS2 (Clavesin 2)	XM_013992600
5.10 2.27E-72	SSTR3 (Somatostatin receptor 3)	NM_001167628
5.09 1.45E-44	TRPM5 (Transient receptor potential cation channel subfamily M member 5)	XM_021082616

A total of 9,511 genes were identified.

**Table 2:** KEGG pathway functional classification of the upregulated genes in islets cultured for a long period of time at 37°C, compared to 24°C Top 20 in terms of *p*-valueTop 20 in terms of gene expression.

Pathway	Count	p-value
**Metabolic pathway**	231	2.10E-16
**Protein processing in endoplasmic reticulum**	45	5.20E-11
**Insulin secretion**	25	3.30E-07
**Retrograde endocannabinoid signaling**	33	3.30E-06
Cardiac muscle contraction	23	7.50E-06
**Dopaminergic synapse**	29	1.30E-05
**Adrenergic signaling in cardiomyocytes**	31	1.50E-05
GABAergic synapse	23	1.60E-05
Maturity onset diabetes of the young	11	5.20E-05
Morphine addiction	22	6.30E-05
Dilated cardiomyopathy	22	1.00E-04
Arrhythmogenic right ventricular cardiomyopathy	19	1.30E-04
**Serotonergic synapse**	25	1.40E-04
Various types of N-glycan biosynthesis	13	1.60E-04
**Glutamatergic synapse**	24	1.90E-04
Amphetamine addiction	17	2.70E-04
Hypertrophic cardiomyopathy	20	4.10E-04
N-Glycan biosynthesis	14	4.10E-04
Cholinergic synapse	23	4.90E-04
Circadian entrainment	21	5.30E-04
**Pathway**	**Count**	**p-value**
**Metabolic pathway**	231	2.10E-16
Pathways of neurodegeneration - multiple diseases	64	1.80E-02
Neuroactive ligand-receptor interaction	49	5.40E-03
Alzheimer disease	48	2.20E-02
**Protein processing in endoplasmic reticulum**	45	5.20E-11
Parkinson disease	37	1.20E-02
Thermogenesis	35	3.70E-07
cAMP signaling pathway	34	3.20E-03
**Retrograde endocannabinoid signaling**	33	3.30E-06
Diabetic cardiomyopathy	33	1.80E-03
Calcium signaling pathway	33	2.30E-02
**Adrenergic signaling in cardiomyocytes**	31	1.50E-05
**Dopaminergic synapse**	29	1.30E-05
Oxytocin signaling pathway	27	8.20E-04
Cell adhesion molecules	27	1.50E-03
Axon guidance	26	2.50E-02
**Insulin secretion**	25	3.30E-07
**Serotonergic synapse**	25	1.40E-04
cGMP-PKG signaling pathway	25	1.70E-02
**Glutamatergic synapse**	24	1.90E-04

Top 20 in terms of gene expression.

A total of 9,511 genes were identified. The genes in bold were in the top 20 for both p-value and abundance.

**Figure 8:**
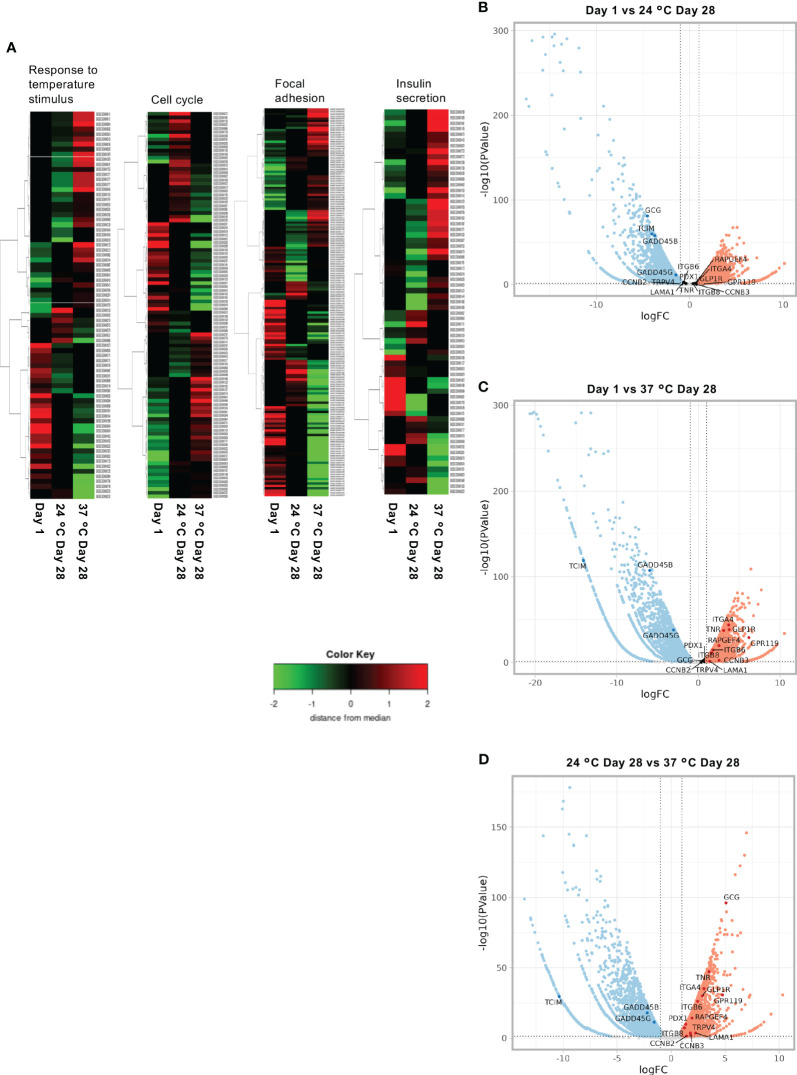
Results of the RNA sequencing of long-term cultured islets. **(A)** Heat map showing the upregulated and downregulated genes involved in the response to temperature stimulus, cell cycle, focal adhesion, and insulin secretion for Day 1, 24°C Day 28, and 37°C Day 28 islets. **(B–D)**. Volcano plots of the gene expression of porcine islets to compare Day 1 and 24°C Day 28 **(B)**, Day 1 and 37°C Day 28 **(C)**, and 24°C Day 28 and 37°C Day 28 **(D)**.

**Figure 8** shows a heat map (**Figure 8A**) and volcano plot (**Figures 8B–D**), which display the up- and downregulated genes for comparisons of the Day 1, 24°C Day 28, and 37°C Day 28 groups. Islets cultured for 28 days at 37°C showed differences in the expression of genes involved in the repression of immunity (*Tcim*), temperature-dependent insulin secretion (*Trpv4*), cell cycle promotion (*Gadd45b*, *Gadd45g*, *Ccnb2*, and *Ccnb3*), adhesion and the ECM (*Tnr*, *Itgb8*, *Itgb6*, *Itga4*, and *Lama1*), and endocrine function and pancreatic differentiation (*Glp1r*, *Rapgef4*, *Gpr119*, *Pdx1*, and *Gcg*).

### 3.7 The effects of the transplantation of islets cultured long-term at 37°C were not inferior to those of overnight-cultured islets

**Figure 9:**
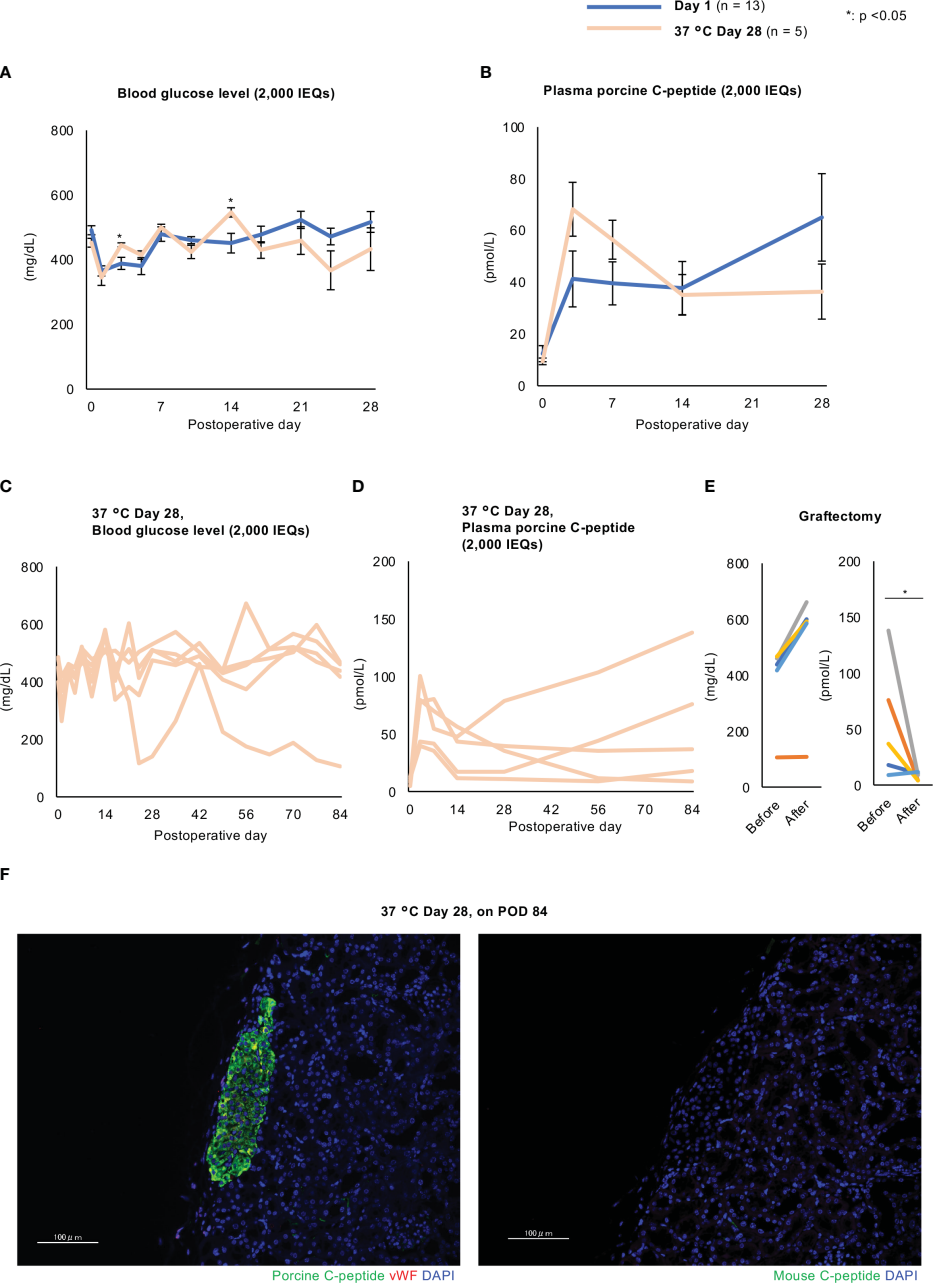
Effects of the xenotransplantation of long-term cultured islets into diabetic nude mice. **(A, B)** Blood glucose **(A)** and plasma porcine C-peptide **(B)** concentrations in diabetic nude mice after the xenotransplantation of porcine islets cultured for 1 day (Day 1 group, blue) or 28 days (37°C Day 28 group, pale orange) (2,000 IEQs) at 37°C. C and **(D)** Individual data for mice transplanted with 37°C Day 28 islets. Blood glucose **(C)** and plasma porcine C-peptide **(D)** concentrations. **(E)** Blood glucose (left) and plasma porcine C-peptide (right) concentrations before and after graftectomy. **(F)** Transplanted porcine islets cultured for 28 days at 37°C and examined 84 days after transplantation, immunostained for porcine C-peptide (green, left) or mouse C-peptide (green, right) and von Willebrand factor (red), and counterstained with DAPI (blue). Scale bar: 100 µm. * *p* < 0.05.

Finally, we performed islet xenotransplantation into diabetic nude mice using long-term cultured porcine islets. The number of transplanted islets was 2,000 and 4,000 IEQs (purity: over 90%, tissue volume: less than 100 µL per 2,000 IEQs). Two thousand IEQs is considered as the minimum number which enables to improve blood glucose. The blood glucose concentrations of the mice were not normalized when they were transplanted with islets cultured for 1 day or 28 days at 24°C (**Supplemental Figures 6A, B**), but their plasma porcine C-peptide concentrations gradually increased with time and the transplanted islets were found to have successfully engrafted in both groups (**Supplemental Figures 6C–F**). The plasma concentrations of porcine C-peptide in the 24°C Day 28 group were significantly lower than those in the Day 1 group (Supplemental **Figures 6C, D**). However, the transplantation efficacy of islets cultured long-term at 37°C was similar to that of overnight-cultured islets. The blood glucose concentrations were similar in the Day 1 and 37°C Day 28 groups (**Figure 9A**) and their plasma porcine C-peptide concentrations increased after transplantation to similar levels (9.44 ± 1.28 pmol/L at transplantation vs. 55.50 ± 21.13 pmol/L 84 days later, *p* = 0.04; **Figure 9B**). Nevertheless, four of the five mice in the 37°C Day 28 group failed to return to normoglycemia (**Figure 9C**). The one mouse that became normoglycemic did not show an increase in blood glucose concentration following graftectomy (**Figure 9E**). In contrast, an increase in plasma porcine C-peptide concentration occurred in all the mice (**Figure 9D**), and this decreased in four out of five of the mice in the 37°C Day 28 group following graftectomy (55.50 ± 21.13 pmol/L before graftectomy vs. 7.79 ± 1.41 pmol/L after graftectomy, *p* = 0.04; **Figure 9E**). The transplanted islets cultured for 28 days at 37°C remained engrafted 3 months after xenotransplantation (**Figure 9F**). Thus, 37°C is a more effective culture temperature than 24°C for the long-term culture of porcine islets to be used in xenotransplantation. Thus, the transplantation efficacy of long-term cultured islets at 37°C was not inferior to that of overnight-cultured islets.

## 4 Discussion

The xenotransplantation of porcine organs, including porcine islets, may be a feasible therapeutic approach in the future. For the promotion of porcine islet xenotransplantation, further innovation is needed to prolong the lifetime of grafts. The identification of the optimal long-term culture temperature for porcine islets represents a substantial challenge to successful porcine islet xenotransplantation because high-quality porcine islets are required. Extensive research has been carried out on the long-term culture of porcine islets, but the characteristics and function of the islets and the optimal temperature for such culture remain a subject for discussion. For example, Brandhorst and colleagues found that 37°C was the optimal temperature for the maintenance of insulin secretion, but it was not suitable for the preservation of cell number (16). Krickhahn and colleagues showed that porcine islets were significantly attenuated in function after 11 days of culture at 24°C (36). However, Rijkelijkhuizen and colleagues achieved a 5-month period of survival of grafts in diabetic rats that were derived from porcine islets cultured at 37°C for 1.5–3 weeks (37).

In the present study, we have assessed the effects of long-term (28-day) culture on porcine islets at either 24°C or 37°C. The long-term culture did not affect the viability of the islets. Furthermore, we made three findings relevant to the establishment of the optimal long-term culture conditions. Firstly, long-term culture at 37°C promoted the morphological stability of the islets. In general, porcine islets are fragile and are considered not to be suitable for long-term culture. We found that most of the islets cultured at 37°C became solid and compact, with a smooth surface, between days 7 and 14, whereas most of them cultured at 24°C had rough, frayed surfaces. We hypothesized that this stabilization was provided by a strengthening of cell-to-cell junctions, secondary to the proliferation of ECM and adhesion factors, and we found that the expression of collagen I and integrin β1 in the cell membranes of porcine islets is significantly increased by long-term culture at 37°C. RNA sequencing of islets cultured long-term at 37°C also showed the upregulation of genes involved in ECM and adhesion. These increases in expression might be responsible for the strengthened cell-ECM junctions, and therefore contribute to the stability of the islets. This strengthening might also be responsible for the improvement in insulin production and secretion in long-term cultured porcine islets (38).

Second, long-term culture at 37°C promoted the proliferation of cells within the cultured porcine islets, which caused an increase in islet size. Ki67-positive islet cells, including both β and non-β cells, were significantly more numerous in the islets following long-term culture at 37°C. Furthermore, RNA sequencing revealed that this culture temperature promoted cell cycle progression (downregulation of *Gadd45b* and *Gadd45g*, upregulation of *Ccnb2* and *Ccnb3*) (39). These data indicate that long-term culture at 37°C promotes the proliferation of endocrine cells by activating the cell cycle. We also consider that the increase in islet size was the result not only of cellular proliferation but also of endocrine differentiation from pancreatic progenitors. The long-term cultured islets were found to contain CD146-positive cells, which are considered to be PSCs, and some of the CD146-positive cells were also found to express PDX-1.

Third, long-term culture at 37°C led to a recovery of the endocrine function of long-term cultured islets. Although long-term culture attenuated the endocrine function of porcine islets, assessed using GSIS and insulin content, this change was mitigated by culture at 37°C. Furthermore, the expression of genes involved in pancreatic regeneration and encoding hormones was higher when long-term culture was performed at 37°C. RNA sequencing analysis also revealed that the expression of the genes involved in insulin secretion, positive regulation of the cAMP signaling pathway, and calcium homeostasis was upregulated when the islets were cultured at 37°C, which may increase insulin secretion. This might be explained by activation of the thermosensitive system controlled by TRP channels, and the expression of TRPM5 was found to be upregulated after culture at 37°C. This encodes a Ca^2+^-activated cation channel that is activated at 15–35°C (40). Previous studies have shown that TRPM5 is expressed in islets, where it regulates the frequency of Ca^2+^ oscillations and contributes to insulin secretion (41–43).

We consider that PSCs, which are CD146-positive cells, were the principal contributors to the morphological change, cellular proliferation, and recovery of endocrine function during long-term culture at 37°C. PSCs are minor cellular components of the periacinar, perivascular, and periductal spaces in the pancreas (44, 45), and previous studies have revealed the roles of PSCs in the synthesis of various ECM proteins, such as procollagen III, collagen I, laminin, and fibronectin (46), which maintain the periinsular basement membrane (47). PSCs are also a key player in the fibrosis that occurs in chronic pancreatitis and pancreatic cancer (48, 49). PSCs also contribute to cellular proliferation through paracrine effects (50, 51). Furthermore, PSCs are multipotent cells that can differentiate into insulin-producing cells (25). Recently, Paul and colleagues assessed whether co-culture with PSCs improves the viability and function of porcine islets. They found that islets co-cultured with PSCs at 37°C showed less fragmentation and disaggregation, higher viability, greater insulin and glucagon production, higher PDX-1 expression, and superior GSIS (52). In the present study, we found that long-term culture at 37°C promoted the proliferation of spindle-like cells derived from islets that attached to the culture vessels. Although this caused the trapping of the islets, the attached cells included multipotent stem cells, which are considered to be CD146-positive PSCs. CD146-positive cells were significantly more abundant in islets cultured at 37°C and might contribute to the superior stability, cellular proliferation, and endocrine function of the islets via paracrine effects and pancreatic differentiation.

Regulation of immunity is also essential for success of xenotransplantation, as same as allogeneic transplantation. However, further knowledges about mechanism of rejection against xenograft are necessary for the success (53). Three predominant carbohydrate antigens, α-Gal, New5Gc and SDa, are the targets for rejection of xenograft. Human naturally harbor antibodies against the antigens in serum. In hyperacute rejection, the antibodies induce complement activation via classical pathway at porcine xenotransplantation. The activated complements injure xenogeneic endothelial cells (54). Binding of the antibodies also induce activation of xenogeneic endothelial cells, which might cause intravascular thrombosis (55). In antibody-mediated rejection, activated B cells and CD4^+^ T cells via presentation of xenoantigens by antigen-presenting cells (APCs) attack xenograft by production of xenoantigen-specific antibodies (56, 57). And in cellular rejection, the rejection is induced by activated CD4^+^T cells and CD8^+^T cells through xenoantigen presented by donor APCs via SLA and recipient APCs via human leukocyte antigen (9, 58, 59). In this study, we attempted to elucidate the immunogenicity of porcine islet under long-term culturing. Interestingly, immunofluorescence and flow cytometry provided no evidence of α-Gal expression in the porcine islets. The α-Gal epitope is a porcine-specific carbohydrate that is a mediator of hyperacute rejection in pig-to-human xenotransplantation (60). Indeed, 70%–90% of human antibodies target the α-Gal epitope (61). Therefore, the regulation of α-Gal is essential for successful heart and kidney xenotransplantation (62, 63). Bottino and colleagues revealed that over 8 months of graft survival could be achieved using islets from pigs with disruptions to their α1,3-galactosyltransferase genes (GTKO pig) with anti-CD154 antibody (64). However, we consider that manipulating α-Gal might not be necessary for successful islet xenotransplantation, in contrast to the requirement for successful organ transplantation. Other molecules might have a larger role in the success of the islet xenotransplantation, but further studies are recommended.

We selected 2 – 3 years-old adult pigs as donors for islets in this study. On the other hand, some groups showed superiorities of neonatal porcine islets in easiness of islet isolation and reverse of diabetes in transplantation (65, 66). Indeed, age is an important factor in deciding suitable donor. Regarding procedure of islet isolation, the procedure for neonatal pigs is easy and inexpensive similar to rodent islet isolation. In a study by Korbutt et al., pancreas acquired from 1 - 3 day-old neonatal pigs with 1.5 ~ 2.0 kg body weight are minced to 1 ~ 2 mm^3^ size. They were transferred to a collagenase solution and gently shaken in a water bath at 37°C for 16 ~ 18 minutes. After filtrating in a 500 um pore-size mesh filter and washing with a buffer solution, the digestion was cultured in Petri dishes. A purification process before a culture is not necessary. Approximately 50,000 islet equivalents (IEQs) can be obtained by this method (67). Unfortunately, neonatal islets harbor some disadvantages in islet yield and immaturity. Published islet yield from fetal and neonatal pancreases were ~ 8,000 IEQs and 20,000 ~ 50,000 IEQs, respectively (68–71). It might be difficult to improve patients with diabetes with that number. Regarding the immaturity of the islets, the therapeutic effect of transplantation to diabetic nude mice was delayed in neonatal islets (6 ~ 10 weeks) (72). Furthermore, the expression of α-Gal in fetal and neonatal islets is stronger than in adult islets (73). In contrast, adult pigs have a larger number of mature islets (68, 69, 71, 74). The isolation procedure is difficult and expensive, although the concept is the same as neonatal islet isolation. The adult porcine pancreas is larger than a neonatal porcine pancreas. More expensive materials are needed, including collagenase, washing buffer, density gradient solution, cold preservation solution for procurement and culture medium, and perfusion, digestion, and purification equipment are required for adult porcine islet isolation. And as previously mentioned, the fragility of adult porcine islets adds to the difficulty (75). Particularly, young adult porcine islets are more fragile than older porcine islets (75). According to Buhler and colleagues, islet capsules could not be found in young porcine islets (76). Meyer and colleagues found that the expressions of collagen I, III, and IV in peri-islet are higher in older pigs than in younger pigs (77).

In this study, we focused on the influence of culture temperature for long-term culture of porcine islets. However, many issues should be considered for establishment of the optimal condition of long-term culture, including oxygenation, composition of culture medium, 2D or 3D culture. Among them, size of islets might be critical for the success of long-term culture. It is obvious for the success of islet transplantation to transplant high volume of islets, i.e. high IEQs. IEQs are influenced by the number of larger islets. However, there are no correlations between size and function of islets. For example, large islets are sensitive to hypoxia. Necrosis is frequently seen at the center of large islets by hypoxia during the culture (78). Furthermore, there are differences in endocrine cell composition among the size of islets. While small islets harbor a lot of β cells, the population of α and δ cells is increased in large islets, especially human islets (79). Therefore, reconstruction of islets in uniform size might be considerable issue for long-term culture of porcine islets.

The present study had two principal limitations. The first was the sample size. The main reason for the small sample size was the difficulty of guaranteeing the quality of the porcine islets, owing to the technical difficulty of islet isolation. The second was a failure of the recipient mice to achieve normoglycemia following transplantation. Porcine islets are fragile, and therefore it might be difficult to prepare recipient animals appropriately for transplantation. In addition, the difference in pig and mouse insulin might have influenced the transplantation efficacy. However, we have shown that 37°C is superior for the successful long-term culture of porcine islets and the mechanism involved. The loss of islets and functional impairment vs. fresh islets are the key challenges to the successful use of long-term culture. Alternative culture methods, including perfusion culture and large-scale three-dimensional culture, should be evaluated in the future for clinical use.

**Figure 10:**
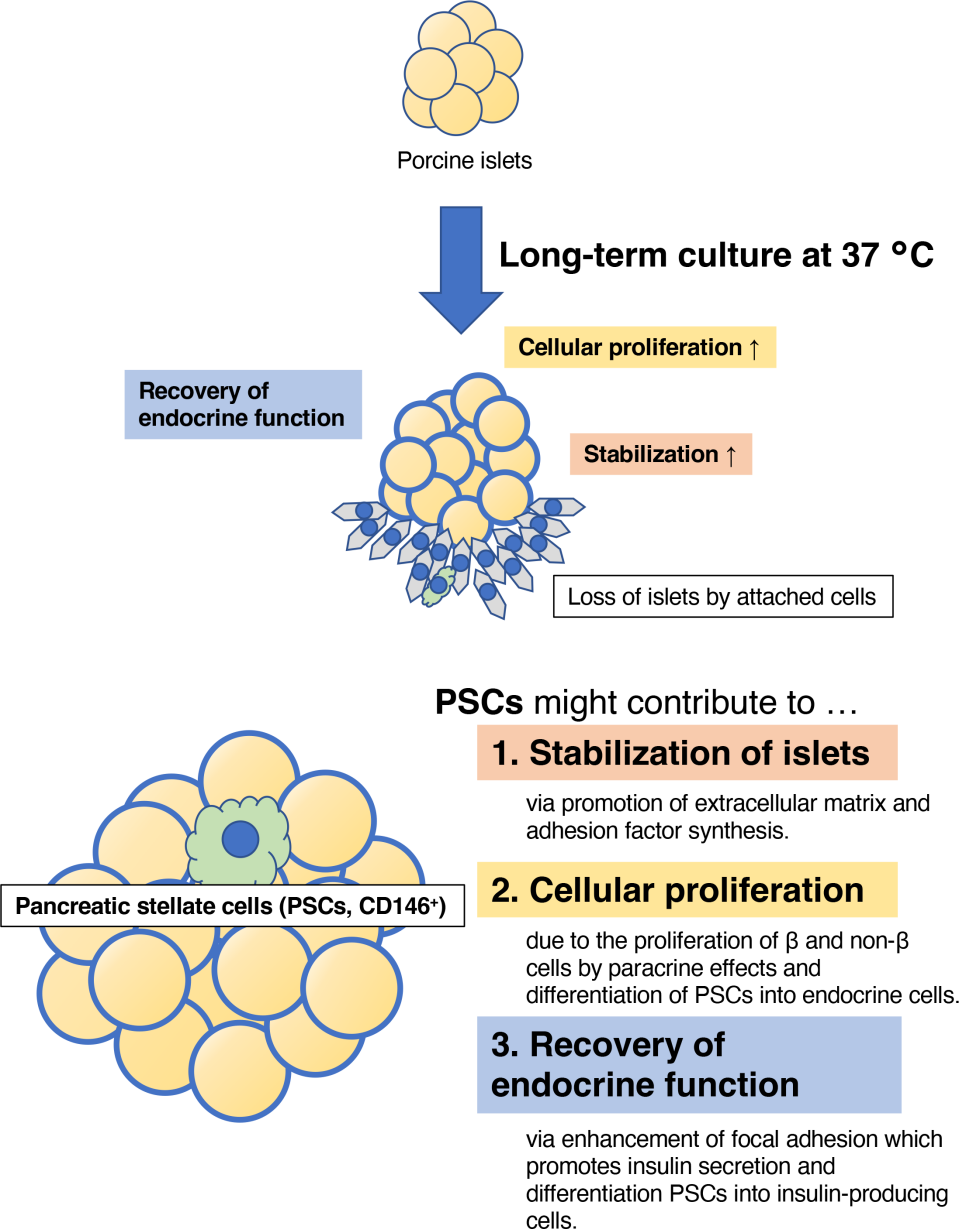
The estimated therapeutic effects of long-term culture on porcine islets. Culture at 37°C contributed to the stability of the morphology of the islets, the proliferation of islet cells, and the recovery of endocrine function, indicated by the expression of genes involved in pancreatic development, hormone production, and glucose-stimulated insulin secretion. These advantages may be provided by islet-derived CD146-positive stellate cells.

In conclusion, we have assessed the optimal temperature for the long-term culture of porcine islets, and found that a temperature of 37°C provides some benefits in better stability, cellular proliferation, and the recovery of insulin secretion in culture (**Figure 10**). Therefore, 37°C might be a suitable temperature for the long-term culture of porcine islets, but further modifications will be required for successful xenotransplantation in a clinical setting.

## Data availability statement

The original contributions presented in the study are publicly available. This data can be found here: DOI: 10.6084/m9.figshare.24203670.

## Ethics statement

The animal study was approved by the Animal Care and Use Committee of Fukuoka University. The study was conducted in accordance with the local legislation and institutional requirements.

## Author contributions

NS: conceptualization, data curation, formal analysis, funding acquisition, investigation, writing – original draft. GY: investigation, methodology, writing – review & editing. RK: investigation, methodology, writing – review & editing. CA: investigation, methodology, writing – review & editing. SK: investigation, methodology, supervision, writing – review & editing.
