# Supplementary figures and images for: Optimal temperature for the long-term culture of adult porcine islets for xenotransplantation
Source: Front Immunol. 2023 Oct 13;14:1280668. doi: 10.3389/fimmu.2023.1280668 (PMC10611499; doi:10.3389/fimmu.2023.1280668)

# Supplemental Figure 1

\*: p < 0.05  
\*\*: p < 0.01  
triplicated

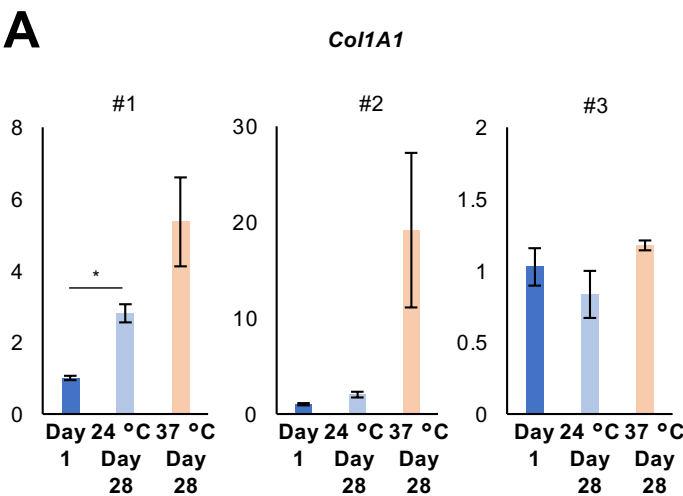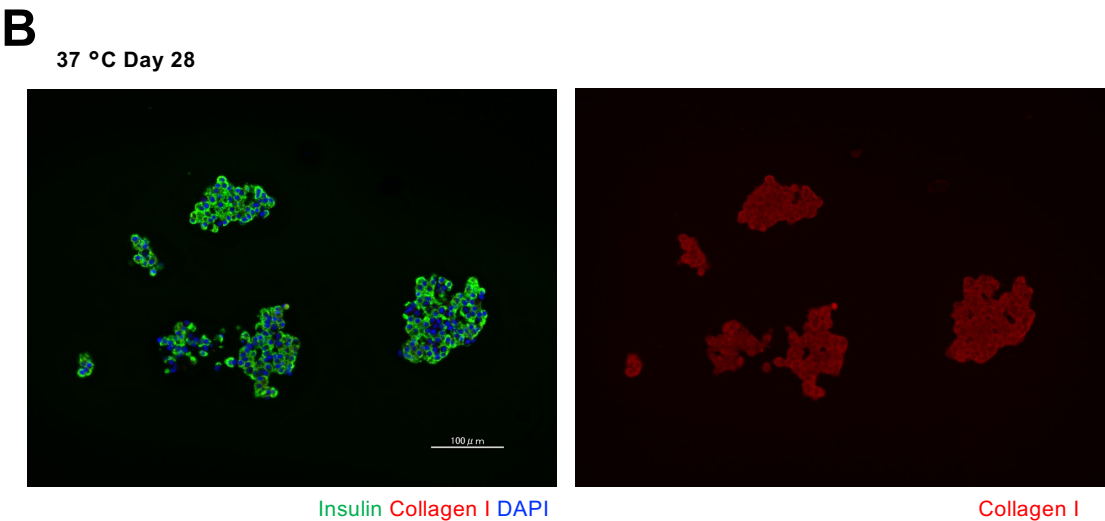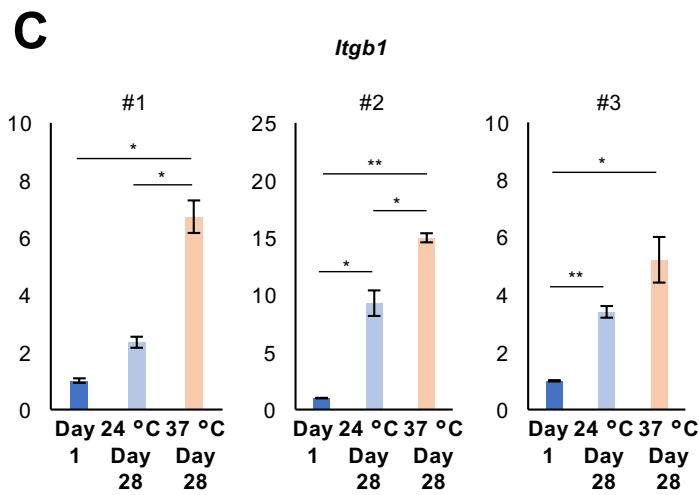

Supplement: Supplementary file 1 [file DataSheet_1.pdf]

# Supplemental Figure 2

\*: p <0.05

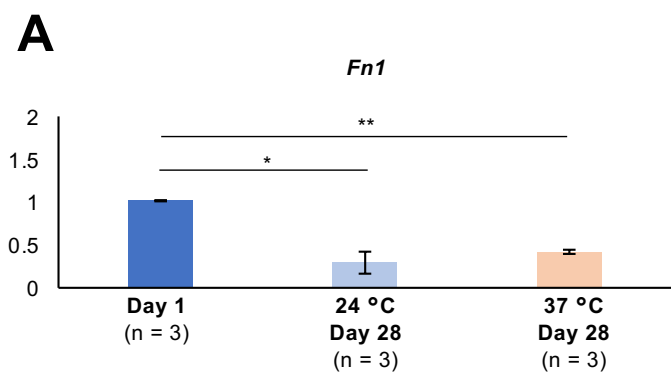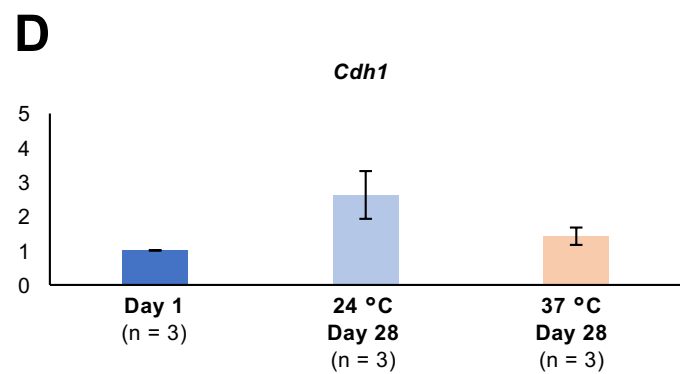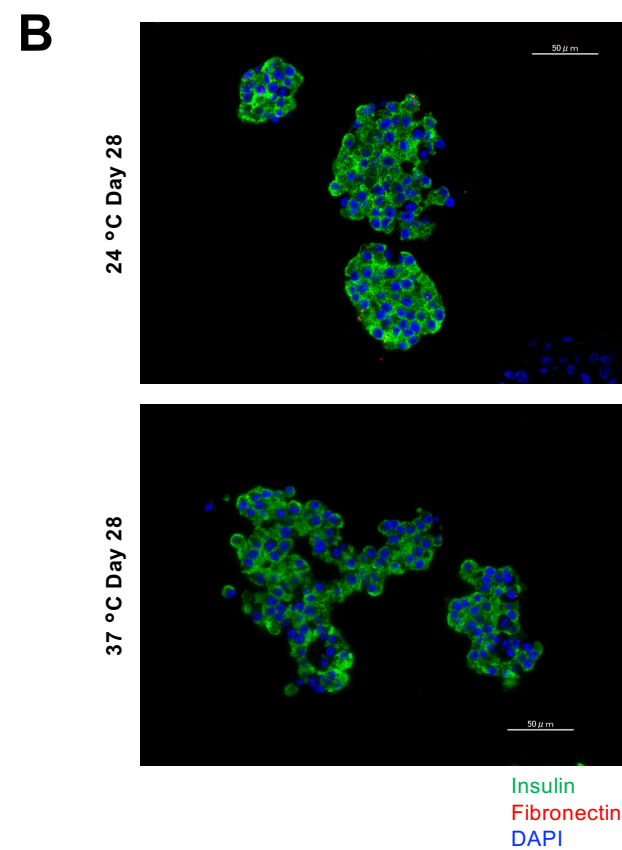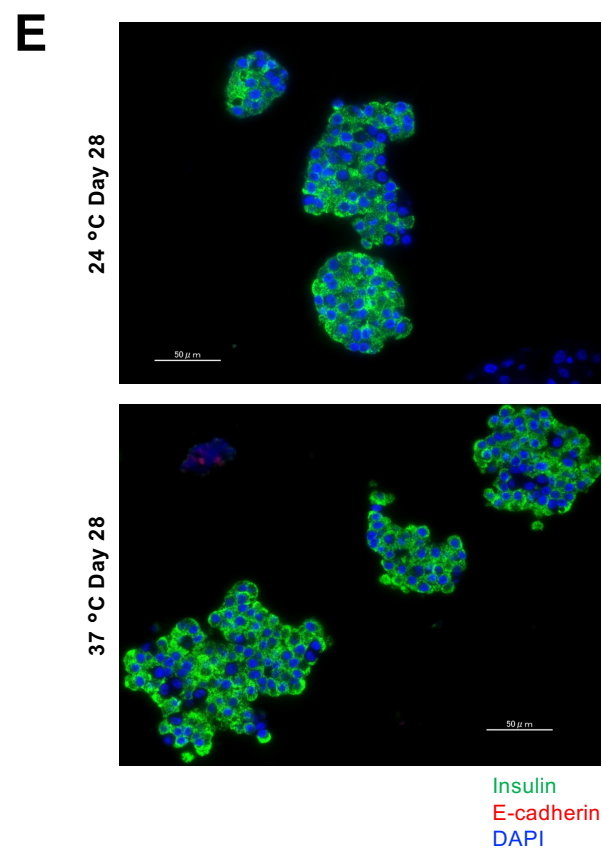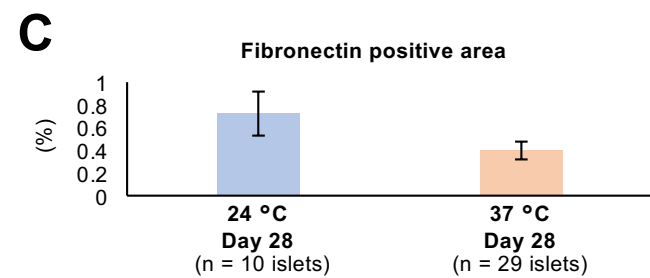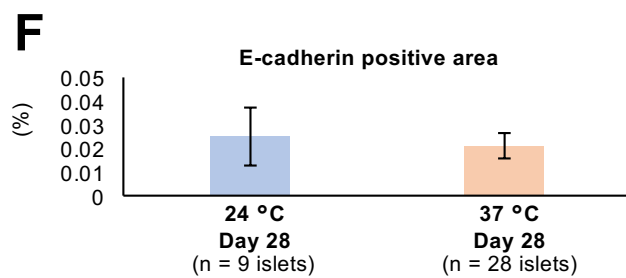

Supplement: Supplementary file 2 [file DataSheet_2.pdf]

# Supplemental Figure 3

\*:  $p < 0.05$   
 \*\*:  $p < 0.01$   
 \*\*\*:  $p < 0.001$   
 triplicated

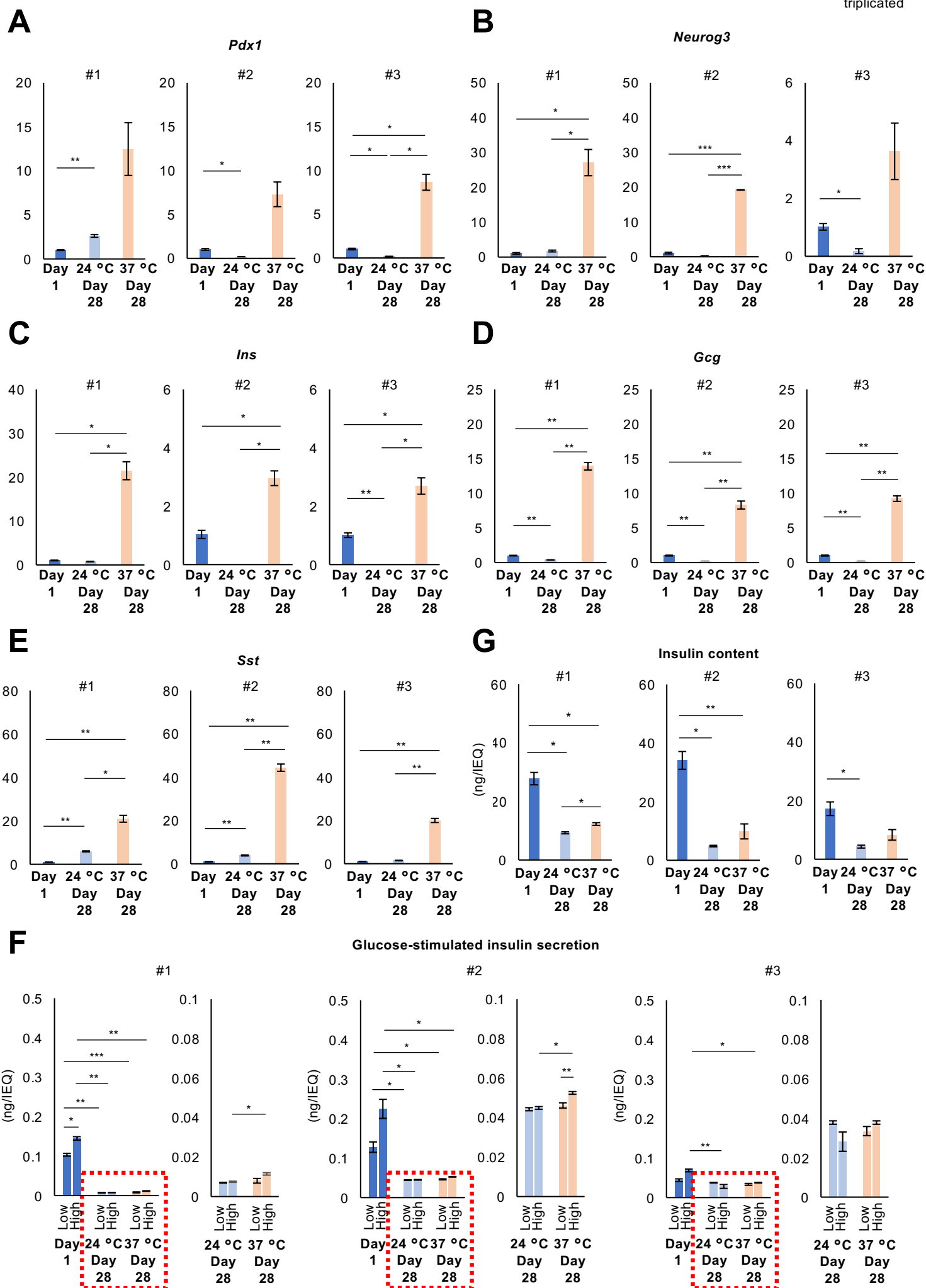

Supplement: Supplementary file 3 [file DataSheet_3.pdf]

# Supplemental Figure 4

A

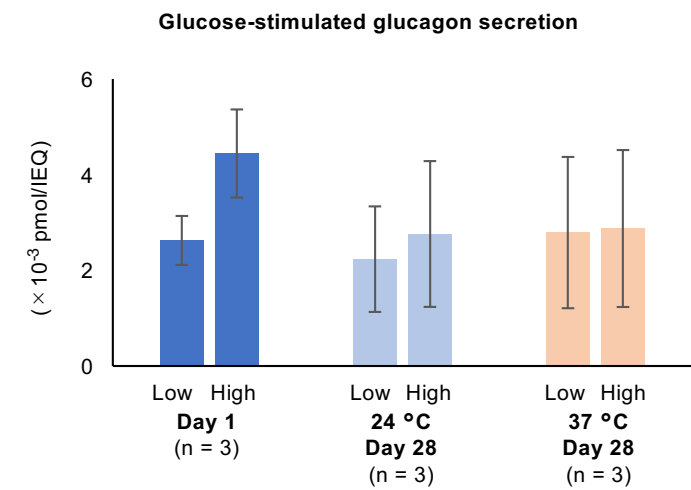

B

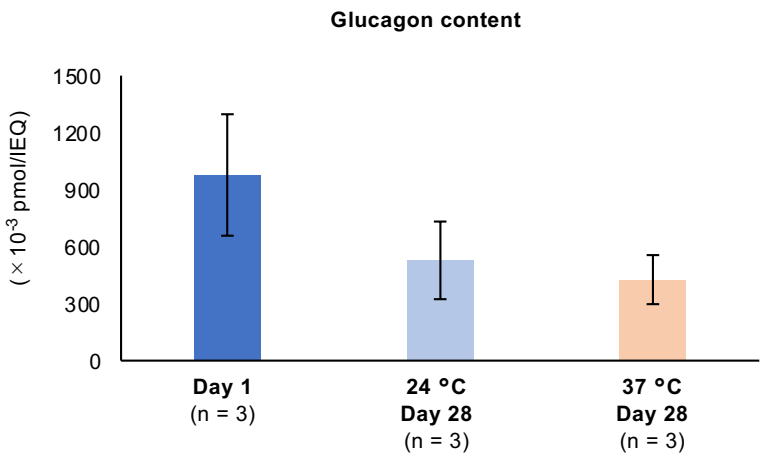

Supplement: Supplementary file 4 [file DataSheet_4.pdf]

# Supplemental Figure 5

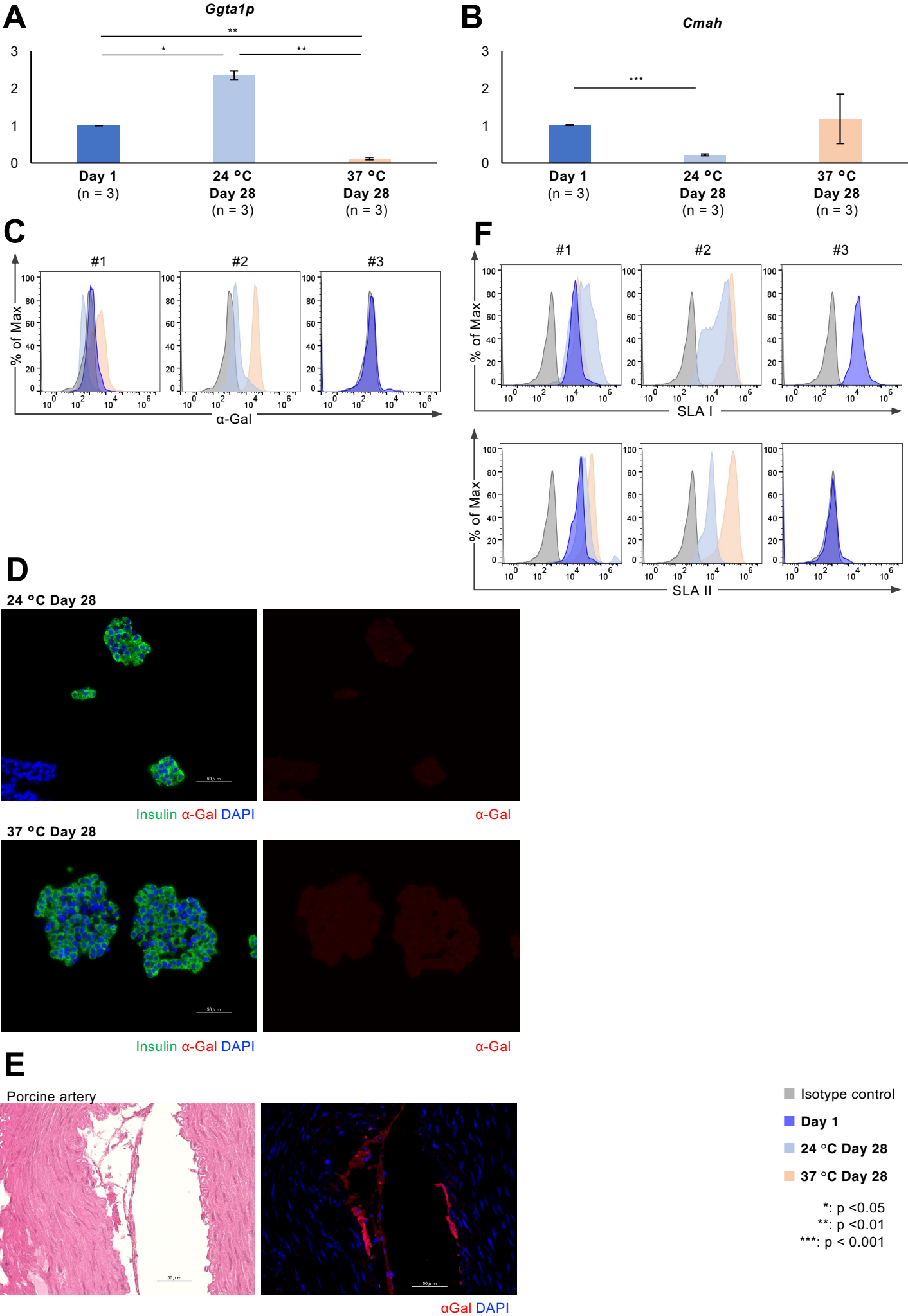

Supplement: Supplementary file 5 [file DataSheet_5.pdf]

# Supplemental Figure 6

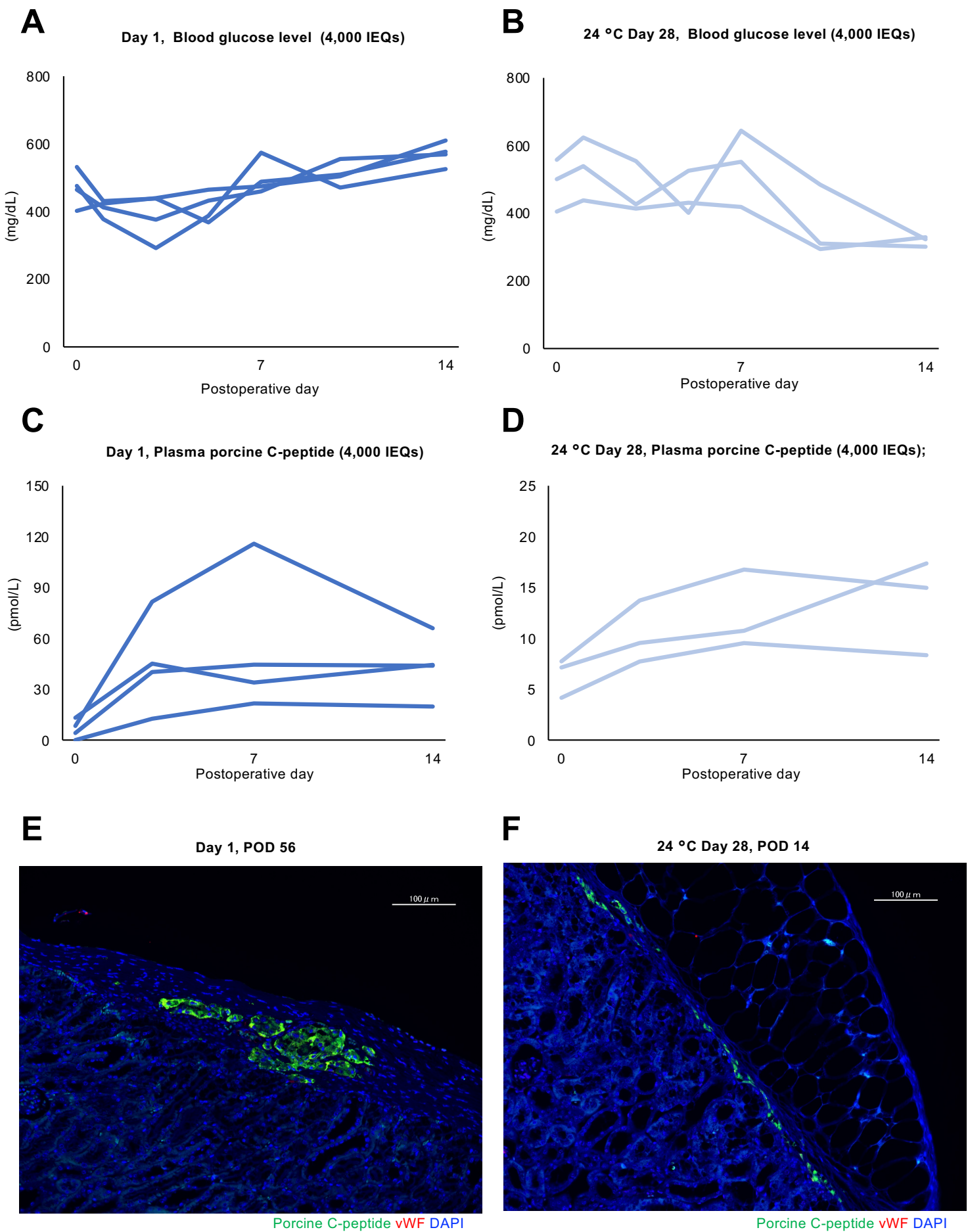

Supplement: Supplementary file 6 [file DataSheet_6.pdf]
