# Supplementary material for: Optimal temperature for the long-term culture of adult porcine islets for xenotransplantation
Source: Front Immunol. 2023 Oct 13;14:1280668. doi: 10.3389/fimmu.2023.1280668 (PMC10611499; doi:10.3389/fimmu.2023.1280668)
Supplement: Supplementary file 7 [file DataSheet_7.docx]

Supplementary Material

# Supplementary Figure Legends

**Supplemental Figure 1. Expressions of collagen I and integrin β1 in long-term cultured porcine islets.**

**A** and **C**. Expressions of *Col1a1* (**A**) and *Itgb1* (**C**) in cultured porcine islets in each islet isolation. We performed three islet isolations for qPCR. #1 - #3 means islet isolation number 1 to 3. The qPCR was performed by triplicate sample analysis. The ratios of the expression between Day 1 and other culture conditions (24°C Day 28 and 37°C Day 28), quantified using the 2^−ΔΔCt^ method. **B**. Histology of isolated islets which were cultured for a long period of time at 37°C. Sections are immunostained with anti-insulin (green) and anti-collagen I (red in B). DAPI (blue) counterstaining for nuclei was used. * *p* < 0.05, ** *p* < 0.01. Scale bar: 100 µm.

**Supplemental Figure 2. Expressions of fibronectin and E cadherin in long-term cultured porcine islets.**

**A** and **D**. Expression of *Fn1* (**A**) and *Cdh1* (**D**). *Fn1* and *Cdh1* encode fibronectin and E-cadherin, respectively. Ratios of the expression on Day 1 and under other culture conditions (24°C Day 28 and 37°C Day 28), shown as 2^−ΔΔCt^ values. n = 3 islet isolations. **B** and **E**. Images of long-term cultured islets at 24°C (upper) or 37°C (lower), and immunostained for insulin (green), fibronectin (red in **B**), and E-cadherin (red in **E**). **C** and **F**. Fibronectin (**C**) and E-cadherin (**F**)-positive areas per islet. DAPI (blue) was used for counterstaining. * *p* < 0.05. Scale bar: 50 µm.

**Supplemental Figure 3. Pancreatic differentiation and endocrine function of islets cultured for a long period of time.**

**A**–**E**. Expression of genes involved in pancreatic differentiation (**A**: P*dx1*, **B**: *Neurog3*) and encoding hormones (**C**: *Ins*, **D**: *Gcg*, **E**: *Sst*) in cultured porcine islets in each islet isolation. Day 1: blue, 24°C Day 28: pale blue, and 37°C Day 28: pale orange. The ratios of the expression after 1 day and 28 days (both temperatures) are shown as 2^−ΔΔCt^ values. **F**. Glucose-stimulated insulin secretion (GSIS) by cultured islets in response to low and high glucose stimulations in each islet isolation. **G**. Insulin content per islet in each islet isolation. We performed three islet isolations for qPCR, GSIS and insulin content. #1 - #3 means islet isolation number 1 to 3. These assessments were performed by triplicate sample analysis. * *p* < 0.05, ** *p* < 0.01, *** *p* < 0.001.

**Supplemental Figure 4. Glucose-stimulated glucagon secretion and glucose content of long-term cultured islets.**

**A**. Glucose-stimulated glucagon secretion of cultured islets in response to low and high glucose concentrations. **B**. Glucagon content per islet. Day 1 (blue), 24°C Day 28 (pale blue), and 37°C Day 28 (pale orange). n = 3 islet isolations.

**Supplemental Figure 5. α-Gal expression in long-term cultured islets.**

**A** and **B**. Expression of genes encoding carbohydrate antigens (**A**: *Gfta1p* and **B**: *Cmah*) in long-term cultured islets. n = 3 islet isolations. **C**. Numbers of α-Gal-positive cells in islets after culture for 1 day, at 24°C for 28 days, and 37°C for 28 days, determined using flow cytometry in each islet isolation. #1 - #3 means islet isolation number 1 to 3. **D**. Long-term cultured islets (upper: 24°C, lower: 37°C) immunostained for α-Gal (red) and insulin (green), and counterstained using DAPI (blue). Scale bar: 50 µm. **E**. Immunofluorescence staining of abdominal artery for α-Gal. Left panel: H&E staining of the artery. Right panel: α-Gal (red). DAPI (blue) was used for counterstaining. Scale bar: 50 µm. **F**. Results of the flow cytometry analysis of islet cells for swine major histocompatibility complex proteins. SLA class I (upper) and SLA class Ⅱ DQ (lower)-positive cells in islets in each islet isolation (Day 1, 24°C Day 28, and 37°C Day 28). #1 - #3 means islet isolation number 1 to 3. * *p* < 0.05, ** *p* < 0.01, *** *p* < 0.001.

**Supplemental Figure 6. Results of the xenotransplantation of long-term cultured islets into diabetic nude mice.**

Four thousand porcine islets were xenotransplanted to diabetic nude mice. **A** and **B**. Blood glucose concentrations of individual mice that underwent the xenotransplantation of porcine islets cultured overnight (Day 1, blue; **A**) and long-term cultured at 24°C (24°C Day 28, pale blue; **B**). **C** and **D**. Plasma porcine C-peptide concentrations of individual mice (Day 1, blue; **C** and 24°C Day 28, pale blue; **D**). **E** and **F**. Histological assessment of engrafted porcine islets (Day 1; **E** and 24°C Day 28; **F**). Double fluorescence-stained for porcine C-peptide (green) and von Willebrand factor (red) was done. DAPI (blue) was used for counterstaining. Scale bar: 100 µm.

# Supplementary Tables

**Supplemental Table 1. Characteristics of the donor microminipigs and isolated islets.**

|  | **P112** | **P114** | **P116** | **P117** | **P118** | **P119** | **P120** |
| --- | --- | --- | --- | --- | --- | --- | --- |
| **Body weight** (kg) | 28.2 | 24.8 | 30.0 | 27.0 | 27.0 | 22.0 | 28.4 |
| **Trimmed pancreas weight** (g) | 69 | 69 | 66 | 78 | 85 | 72 | 49 |
| **Warm ischemic time** (min) | 0 | 0 | 0 | 0 | 0 | 0 | 0 |
| **Cold ischemic time** (min) | 122 | 104 | 124 | 110 | 147 | 104 | 125 |
| **Islet yields after purification** (IEQs) | 109,349 | 99,974.5 | 98,736.5 | 241,354 | 9,208 | 54,965 | 73,329 |
| **Islet yields after purification** (islet numbers) | 118,000 | 209,000 | 263,500 | 314,000 | 43,050 | 160,000 | 99,000 |
| **IEQ / islet number after purification** | 0.93 | 0.48 | 0.37 | 0.77 | 0.21 | 0.34 | 0.74 |
| **Purity after purification** (%) | 90 | 90 | 95 | 95 | 95 | 90 | 95 |
| **Islet quality score after purification** (0-10) | 10 | 9 | 7 | 10 | 6 | 7 | 10 |

**Supplemental Table 2. Porcine primers for real-time reverse transcription-polymerase chain reaction analysis.**

| Primer name | Sequence (5′-3′) | Tm (°C) |
| --- | --- | --- |
| *Actb*_F | CTCCAGAGCGCAAGTACTCC | 60.18 |
| *Actb*_R | TGCAGGTCCCGAGAGAATGA | 60.61 |
| *Ggta1*_F | GAAACCCAGAAGTTGGCAGC | 59.68 |
| *Ggta1*_R | CAGTCCACTAGCGGAAGCTC | 60.18 |
| *Cmah*_F | TCACATGCACTCAGACCACC | 59.96 |
| *Cmah*_R | CAACTGGACGCCACTCTGAT | 60.04 |
| *Ins*_F | GGCTTCTTCTACACGCCCAA | 60.32 |
| *Ins*_R | GCGGCCTAGTTGCAGTAGTT | 60.39 |
| *Gcg*_F | GATCATTCCCAGCTCCCCAG | 59.89 |
| *Gcg*_R | GTGTTCATCAGCCACTGCAC | 59.76 |
| *Sst*_F | CCCGACTCCGTCAGTTTCTG | 60.39 |
| *Sst*_R | GGCATCGTTCTCTGTCTGGT | 59.75 |
| *Pdx1*_F | AAGTCTACCAAGGCTCACGC | 60.04 |
| *Pdx1*_R | GCGCGGCCTAGAGATGTATT | 60.04 |
| *Neurog3*_F | CTCTATCCCTCAGCGCCCTA | 60.25 |
| *Neurog3*_R | CGACGCAGGTCACTTTGTCT | 60.60 |
| *Col1A1_F* | AGCCCTGGTGAAAATGGAGC | 60.61 |
| *Col1A1*_R | CACCCTTAGCACCAACAGCA | 60.54 |
| *Fn1*_F | GCACCATCCAACTTGCGTTT | 59.97 |
| *Fn1*_R | TGTACTCGGTTGCTGGTTCC | 59.97 |
| *Itgb1*_F | GGGACACGCAAGAAAATCCG | 59.83 |
| *Itgb1*_R | TGCTCACTTCCCCTCGTACT | 60.25 |
| *Cdh1*_F | AATGATGTGGCACCAACCCT | 59.89 |
| *Cdh1*_R | TAGCAGCTTCGGAACCACTG | 60.04 |

**
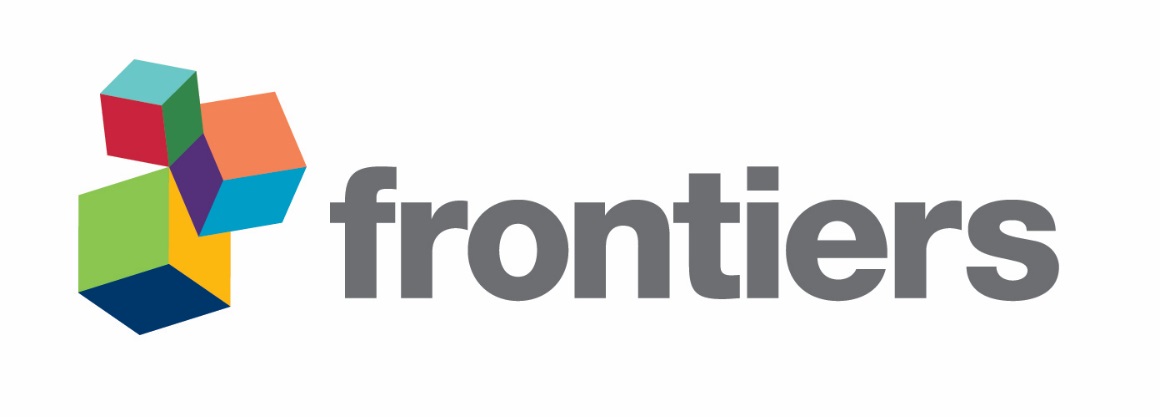
**
